# Supplementary material for: Hierarchical folding and reorganization of chromosomes are linked to transcriptional changes in cellular differentiation
Source: Mol Syst Biol. 2015 Dec 28;11(12):852. doi: 10.15252/msb.20156492 (PMC4704492; doi:10.15252/msb.20156492)
Supplement: Supplementary file 1 — Appendix [file MSB-11-852-s001.pdf]

Appendix
for

Hierarchical folding and reorganization of chromosomes are linked to transcriptional changes during cellular differentiation

James Fraser[1,*], Carmelo Ferrai[2,3,*], Andrea M. Chiariello[4,*], Markus Schueler[2,*], Tiago Rito[2,*], Giovanni Laudanno[4,*], Mariano Barbieri[2], Benjamin L. Moore[5], Dorothee C.A. Kraemer[2], Stuart Aitken[5], Sheila Q. Xie[3,#], Kelly J. Morris[2,3], Masayoshi Itoh[6,7], Hideya Kawaji[6,7], Ines Jaeger[8,‡], Yoshihide Hayashizaki[6], Piero Carninci[7], Alistair R.R. Forrest[7,f], the FANTOM Consortium, Colin A. Semple[5,§], Josée Dostie[1,§], Ana Pombo[2,3,§], and Mario Nicodemi[4,§]

Addresses:

[1]Department of Biochemistry and Goodman Cancer Centre, McGill University, 3655 Promenade Sir-William-Osler, Montréal, Québec, Canada, H3G1Y6.

[2]Epigenetic Regulation and Chromatin Architecture Group, Berlin Institute for Medical Systems Biology, Max-Delbrück Centre for Molecular Medicine, Robert-Rössle Strasse, Berlin-Buch 13125, Germany.

[3]Genome Function Group, MRC Clinical Sciences Centre, Imperial College London, Hammersmith Hospital Campus, London W12 0NN, UK.

[4]Dipartimento di Fisica, Università di Napoli Federico II, and INFN Napoli, CNR-SPIN, Complesso Universitario di Monte Sant'Angelo, 80126 Naples, Italy.

[5]MRC Human Genetics Unit, MRC IGMM, University of Edinburgh, Edinburgh EH4 2XU, UK.

[6]RIKEN Preventive Medicine and Diagnosis Innovation Program, Wako, Saitama, 351-0198 Japan.

[7]RIKEN Center for Life Science Technologies, Division of Genomic Technologies, Yokohama, Kanagawa, 230-0045 Japan

[8]Stem Cell Neurogenesis Group, MRC Clinical Sciences Centre, Imperial College London, Hammersmith Hospital Campus, London W12 0NN, UK.

[#]Present address: Single Molecule Imaging Group, MRC Clinical Sciences Centre, Imperial College London, Hammersmith Hospital Campus, London W12 0NN, UK.

[‡]Present address: Cardiff School of Biosciences, Maindy Road, Cathays, Cardiff CF24 4HQ, UK.

[f]Present address: Systems Biology and Genomics, Harry Perkins Institute of Medical Research, Nedlands, WA 6009, Australia.

*Equal contribution.

[§]Joint corresponding authors:

Colin A. Semple:	colin.semple@igmm.ed.ac.uk
Josée Dostie:	josee.dostie@mcgill.ca
Ana Pombo:	ana.pombo@mdc-berlin.de
Mario Nicodemi:	mario.nicodemi@na.infn.it

Table of Contents

Supplementary Methods page 3

Supplementary Analyses page 27

Description of Supplementary Tables page 29

Supplementary References page 30

Appendix Figures page 35

Other supplementary material for this manuscript includes the following:

Tables EV1, EV2 and EV3 (Excel format)

Supplementary Methods

Cell culture and neuronal differentiation

Cells were grown at 37°C in a 5% (v/v) CO_2 incubator. Mouse embryonic stem cells ES-46C (Ying et al, 2003) (ESC) were grown in GMEM medium (Invitrogen, # 21710025), supplemented with 10% (v/v) fetal calf serum (FCS; PAA, # A15-151), 2 U/ml LIF (Millipore, # ESG1107), 0.1 mM β-mercaptoethanol (Invitrogen, # 31350-010), 2 mM L-glutamine (Invitrogen, # 25030-024), 1 mM sodium pyruvate (Invitrogen, # 11360039), 1% penicillin-streptomycin (Invitrogen # 15140122), 1% MEM Non-Essential Amino Acids (Invitrogen, # 11140035) on gelatin-coated (0.1% (v/v)) Nunc T25 flasks. The medium was changed every day and cells were split every other day. Before sample collection for Hi-C or CAGE, ESC were plated on gelatin-coated (0.1% (v/v)) Nunc 10 cm dishes in serum-free ESGRO Complete Clonal Grade Medium (Millipore, # SF001-500), containing 1U/ml LIF. Cells were grown for 48h, with a medium change at 24h.

Mouse Epi Stem cells (EpiSC) were established from ESC-46C cells, after growth (4 weeks) in N2B27 basal medium containing 20 ng/ml of Activin (R&D, # 338-AC-050) and 12 ng/ml FGF2 (Peprotech, # 100-18B). The composition of the N2B27 basal medium was: ½ of DMEM/F12 (Invitrogen, # 21331-020), ½ Neurobasal Medium (Invitrogen, # 21103-049), 0.5x N2 (Invitrogen, # 17502-048), 0.5x B27 (Invitrogen, # 12587-010), 0.05 M β-mercaptoethanol (Invitrogen, # 31350-010) and 2 mM L-glutamine (Invitrogen, # 25030-024). EpiSC were grown on Nunc plates coated with FCS (PAA, # A15-151). Culture medium was changed every day and cells were split every other day by washing them 3 times with PBS without magnesium and calcium, incubated in PBS for 3 min at room temperature and then gently scraped from the plate. After scraping them the cells were pipetted up and down 3 times before transferring them to a new FCS-coated plate.

EpiSC cells were differentiated into NPC (Day 5) or Neurons (Day 16) with midbrain properties (Jaeger et al, 2011). Differentiation was optimized based on a previously developed method (Jaeger et al, 2011) and at Day16, 99.4% cells are neurons positive for Tubulin beta-3, and 99.6% negative for BrdU (after 24h incorporation, see below; $n=3.5x10^4$). The day before starting the differentiation protocol (Day '-1'), exponentially growing EpiSC were plated on Nunc plates coated with 15 μg/ml human plasma fibronectin (Millipore, # FC010) and cultured in

N2B27 basal medium containing Activin and FGF2, so that they reached 70-80% confluence after 24h. Differentiation was started by rinsing cells twice with PBS, and culturing in N2B27 basal medium plus 1 μM PD 0325901 (Axon, # 1408) for 2 days. Medium was replenished every day. On day 2, cells were washed with PBS, scraped, replated on 10 cm dishes (Nunc, # 150350) coated with 15 μg/ml human plasma fibronectin (Millipore, # FC010) and cultured in N2B27 basal medium for 3 days. At this stage, medium was replenished every day by removing ½ volume and adding ½ volume of freshly prepared medium. Samples of NPC were collected at this stage at day 5.

Neurons were produced in parallel through the following steps. After 72h, the medium was replaced with N2B27 basal medium plus 100 ng/ml FGF8 (Peprotech, # 100-25-25) and 200 ng/ml Shh (R&D, # 464-sh-025). Medium was replenished every day by removing ½ volume and adding ½ volume of freshly prepared medium.

After 96h, cells were washed with PBS, and the medium replaced with N2B27 basal medium plus 10 ng/ml BDNF (R&D, # 450-02-10), 10 ng/ml GDNF (R&D, # 450-10-10) and 200 μM L-ascorbic acid (Sigma, # A4544). Medium was replenished every day by removing ½ volume and adding ½ volume of freshly prepared medium until the last collection at day 16.

CAGE sample preparation

ESC, NPC and Neurons were grown as indicated above on 10 cm dishes (Nunc, # 150350), the medium was removed, TRIzol (Invitrogen, # 15596-018) was added directly to the dish and total RNA was extracted and purified using the miRNeasy mini kit (Qiagen) from 5x10^6 cultured cells with a column purification according to manufacturer's instructions with modifications. Chloroform was added at 200 μl/ml of TRIzol lysate before centrifugation. To bind total RNA including miRNA to the columns, 1.5x volumes of 100% ethanol was added to the aqueous phase of the lysate. Before library preparation, the quality of RNA samples was checked by BioAnalyzer (Agilent) to ensure integrity of RNA (RIN>8) and final concentration was determined by spectrophotometry (Nanodrop). Samples were sent to Riken Institute, Omics Science Centre (OSC), Yokohama, Japan, for CAGE library preparation and sequencing. CAGE libraries were prepared from 5 μg of total RNA as described previously (Itoh et al, 2012; Kanamori-Katayama et al, 2011) and then sequenced on the HeliScope Single Molecule

Sequencer platform (Helicos Bioscience). The sequencing reads were mapped and aggregated into TSS clusters, as previously described (Forrest et al, 2014).

Incorporation of BrdU in replicating cells

To measure the replication index of the cell populations used, ESC, NPC and Neurons were grown in their normal medium (see above) containing 50 μM BrdU (Sigma, # 59-14-3) for 24 h. Cells were fixed (10 min) with 4% freshly-depolymerized paraformaldehyde in 125 mM HEPES (pH 7.8). After washing (2x) in PBS, cells were permeabilized (20 min) with 0.2% Triton X-100 in PBS and blocked (30 min) with 0.5% BSA, 0.2% fish skin gelatin in PBS. Cells were incubated (1h, humid chamber) with 0.5 U/μl DNaseI (Sigma, # D4263), 3 mM $MgCl_2$ and anti-BrDNA antibody (1:150; raised in mouse; Caltag Laboratories, # MD5110). Cells were washed (3x, 3 min each) with blocking solution and incubated (40-60 min) with the secondary antibody AlexaFluor 555 anti-Mouse IgG (1:1000; raised in donkey; Invitrogen, # A31570), in a humid dark chamber. Cells were washed (2x) with blocking solution and (2x) with PBS. Cells were stained (5 min) in 0.1 mg/ml DAPI in PBS washed (1x) with PBS and mounted using Dako Fluorescence Mounting Medium (Dako Agilent Technologies, # S3023).

Immunofluorescence

ESC, NPC and Neurons were fixed (10 min) with 4% freshly depolymerized paraformaldehyde in 125 mM HEPES, washed twice with PBS and permeabilized (3x, 5 min each) with 0.3% Triton X-100 in PBS (PBST), 5 min each by gently rocking. Cells were blocked (30 min) with 3% donkey serum in PBST and then incubated (at 4°C, overnight), with primary antibody in PBST+3% donkey serum. Primary antibodies used were: mouse anti-Tuj1 (1:500; a Neuronal marker, Sigma, # T8660); mouse anti-Oct4 (1:50; a pluripotency marker; BD Biosciences, # 611202); mouse anti-nestin (1:100; a marker of neural precursor cells, Cell Signalling, # 4760). After washing (3x, 10 min each) with PBST, cells were incubated (1h, in a humid dark chamber) with AlexaFluor 555 anti-mouse IgG (1:1000; raised in donkey; Invitrogen, # A31570) secondary antibodies in PBST+3% donkey serum. Cells were incubated (5 min) in 0.1 mg/ml DAPI in PBST, washed with PBST (3x, 10 min each), before mounting in Dako Fluorescence Mounting Medium.

Images were collected sequentially on a confocal laser-scanning microscope (Leica TCS SP8 STED; HC PLAPOCS2 20x / 0.75 IMM objective), equipped with a 405 diode and a WLL (Supercontinuum visible) laser, and pinhole equivalent to 1 Airy disk. For comparison of BrdU staining during neuronal differentiation, images were collected on the same day using the same settings, and without saturation of the intensity signal.

Generation of Hi-C libraries

Samples used for generating ESC, NPC and Neurons Hi-C libraries were grown as detailed above. The medium was removed from the plate, replaced with fresh serum-free medium containing 1% formaldehyde (Sigma, # F8775), and the samples incubated 10 min at room temperature with gentle rocking every 2 min. Glycine (0.125M final concentration; Sigma; # G8898) was added and mixed by gentle rocking for 5 min to quench the formaldehyde and stop crosslinking. Plates were incubated 15 min on ice to stop the reaction. Cells were scraped from the surface of the Petri dish and the dish was washed thoroughly with media before transferring the cells to a 15 ml conical Falcon tube. Cells were pelleted by centrifugation with a Heraeus Multifuge X3M equipped with a 75003607 rotor (10 min, 2000 rpm), quick-frozen on dry ice and stored at -80˚C.

The initial chromatin digestion, biotin labeling, blunt end ligation, protein removal, DNA purification, and quality control of the initial stage of the Hi-C libraries were all performed exactly as previously published (Lieberman-Aiden et al, 2009), using 400U per reaction of either NcoI or HindIII enzyme. From the 3C-like library, changes were made in order to obtain a higher yield of DNA for sequencing (Appendix Fig S1). The removal of biotin from the ends of unligated restriction fragments was performed by mixing 5 µg of Hi-C DNA, 1 µl of 10x NEB 2 buffer, 1 µl each of 10 mM dATP and 10mM dGTP, and 5U of T4 DNA Polymerase (NEB) in a 200 µl PCR tube (Maxymum Recovery, Axygen). Each reaction was incubated in a PCR machine (Bio-Rad MyCycler) at 12°C for 2h. Up to 10 separate reactions per sample were performed to provide enough DNA for downstream processing, as the percentage of DNA in the library that contains valid, biotin-labeled, Hi-C junctions is very small. The samples were then incubated for 20 min at 75°C to heat-inactivate the enzyme, and were then transferred to 1.5 ml tubes (Maxymum Recovery, Axygen). The DNA was precipitated from the solution by the addition of 10 µl 3M sodium acetate and 275 µl of cold 100% EtOH, followed by incubation overnight at -80°C. Samples were washed 2-3 times with 500 µl of 70% ethanol before being resuspended in

400 µl water. The DNA was then sheared to an average size of 300-500bp using a Branson Sonifier with cup horn attachment (Branson S-450D Sonifier). DNA was then size-selected using Ampure XP beads (Agencourt XP) according to manufacturers specifications, with ratios of 0.65x and 0.8x, and was then eluted in 60 µl of Qiagen EB buffer. All of the replicates for each sample were then pooled together, and the total volume was reduced to 301µl using a speed-vac (Eppendorf). 1 µl of each sample was used to measure DNA concentration using the Quant-iT PicoGreen assay (Invitrogen), where for each sample we would expect to have more than 2 µg of total DNA present.

The biotin-labeled Hi-C fragments were then pulled down to facilitate DNA end repair, adenylation, and adaptor ligation. 60 µl of Dynabeads MyOne Streptavidin C1 beads (Invitrogen) were washed twice with 400 µl of Tween Wash Buffer (TWB; 5 mM Tris-HCl pH 8.0, 0.5 mM EDTA, 1 M NaCl, 0.05% Tween). For all washes, beads were recovered using a DynaMag Magnet (Invitrogen) for 1 min. After washing, the beads were then resuspended in 2x Binding Buffer (BB; 10 mM Tris-HCl, pH 8.0, 1 mM EDTA, 2 mM NaCl). All 300 µl of Hi-C DNA was added to the resuspended beads and incubated with rotation at room temperature for 15 min. The beads were collected, the supernatant discarded, and the Hi-C bound beads were washed with 400 µl of 1x BB, before being transferred to a new 1.5ml tube. The beads were then resuspended in 100 µl of 1x Ligation buffer (NEB) before being transferred into a 200 µl PCR tube.

The ends of the Hi-C fragments were repaired using a solution prepared on ice separately containing 10 µl of 10x Ligation Buffer (NEB), 4 µl 10 mM dNTP mix, 5 µl (15 units) of T4 DNA Polymerase (NEB), 5 µl of T4 Polynucleotide Kinase (NEB), 1 µl of Klenow DNA Polymerase (NEB), and 75 µl of water. The supernatant from the beads was removed immediately prior to being resuspended with the cold 100 µl end repair mix. The beads were then incubated at 20°C for 30 min in a thermal cycler, washed twice with 200 µl of 1x TWB and twice with 200 µl EB buffer. A 3’ adenylation mix was generated in a similar fashion, by preparing separately on ice 5 µl of 10x NEB 2, 10 µl of 1 mM dATP, 3 µl of Klenow exo-, and 32 µl of water. The supernatant from the beads was removed immediately prior to being resuspended with the 50 µl adenylation mix. The samples were incubated at 37°C for 30 min in a thermal cycler, and then washed twice with 200 µl of 1x TWB and twice with 200 µl EB buffer.

The amount of Illumina PE adaptor needed to ligate to the Hi-C fragment ends was determined from the total DNA measured using the Quant-iT kit earlier in the protocol. For every

1 µg of total DNA present in a given Hi-C sample, 6 pmol of the Illumina PE Adaptor was used. On ice, 25 µl of 2x Adaptor Ligation Buffer (Illumina) and the Illumina PE Adaptor was brought to a total volume of 45 µl with water. The beads were then resuspended with the adaptor mix, followed by the addition of 5 µl of DNA ligase (Illumina), and were mixed gently with a pipettor. The ligation reaction was incubated at 20°C for 15 min in a thermal cycler, the beads were washed twice with 200 µl of 1x TWB, twice with 200 µl of EB buffer, and finally resuspended in 50 µl of EB buffer.

PCRs were performed to determine the optimal number of cycles needed to generate enough of a Hi-C library to be sent for sequencing. Individual PCRs were composed of 0.5 µl Hi-C Library, 1 µl of 1.6 µM Illumina PE 1.0 primer, 1 µl of 1.6 µM Illumina PE 2.0 primer, 10 µl of Phusion High-Fidelity 2x Master Mix (NEB), and 7.5 µl water in 200 ml PCR tubes. The reactions were run as follows: 1 cycle of 5 min at 94°C, either 6, 8, 10, 12, or 14 cycles of 30s at 94°C, 30s at 65°C, and 30s at 72°C, and a final cycle of 7 min at 72°C. DNA was then run on a 2.5% agarose gel, where the optimal number of PCR cycles was chosen. This number varied from 7 to 11 cycles for all Hi-C libraries, depending on the quantity of Hi-C DNA initially used. Smaller numbers of PCR cycles are strongly preferred to reduce the abundance of duplicate reads in the data and increasing the yield of the sequencing runs.

A number of large-scale reactions were then performed using 2.5x the reagents from the test PCRs using the chosen cycle number in order to generate enough Hi-C library for sequencing. Samples were purified using a 1.8x volume of Ampure XP beads, as per the manufacturer's instructions, and eluted in 50 µl of EB buffer (Qiagen). Samples were quantified using the Quant-iT assay, and visualized on a 2.5% agarose gel prior to sequencing to check the quantity and quality of the samples.

Sequencing

All Hi-C samples were sequenced on the Illumina Hi-Seq 2000/2500 platform, using the 100bp paired-end protocol (Genome Quebec). Raw reads are available online from GEO, accession number GSE59027. Table EV3 describes the number of reads obtained for each sequenced lane.

Other Hi-C datasets

Raw reads from a similar mouse ESC-J1 Hi-C experiment (Dixon et al, 2012) were downloaded from GEO using accession number GSE35156 and processed in the same manner as our own data for all comparisons in the article. The locations of TADs identified in the same study were also downloaded (HMM-TADs), and are referenced as such in comparisons.

Hi-C Data processing

Hi-C reads were processed using the software presented in Imakaev *et al.* (2012) *hiclib* (Imakaev et al, 2012). Although there are other tools available that deal with biases in Hi-C datasets in a comparable fashion (Hu et al, 2012; Yaffe & Tanay, 2011), *hiclib* has a well documented pipeline that guides analysis from the level of raw reads up to fully corrected Hi-C contact matrices.

Briefly, each Hi-C paired-end read file was aligned separately to the mm9 genome using bowtie2 (Langmead & Salzberg, 2012) using default parameters. Each read was initially truncated to 25bp and then checked to see if it aligned uniquely. If so, it was added to the pool of uniquely mapped Hi-C reads for downstream analysis. If the 25bp read did not align uniquely, the size of the truncated read would be increased by 5bp and another attempt would be made to align it. This is repeated until none of the non-aligning reads can be extended any further. This ensures that the presence of a Hi-C junction within the Hi-C read, often denoting a ligation event between two non-continuous parts of the genome, does not affect the alignment of the read.

Hi-C read pairs that contain two uniquely aligning ends were then parsed and filtered using the default settings of *hiclib* to obtain a collection of high-confidence Hi-C read pairs. Self-circularized products, dangling ends, duplicate reads arising from PCR bias, and reads that align more than 500bp apart through a Hi-C junction were removed from downstream analysis. Reads that map to fragments smaller than 100bp or larger than 100kb were also removed, as were the top 0.5% of fragments with the largest number of reads. Data from different sequencing lanes were combined at this point, and binned using 50kb windows in order to be converted into raw Hi-C contact matrices. Table EV2 details the effects each of the filters had on the quantity of reads. Finally, an iterative *hiclib* correction was applied to normalize the systematic biases in the Hi-C matrices using DNA visibility.

In order to be able to compare data between Hi-C datasets, we used additional methods to normalize across different samples (Appendix Fig S18). The largest source of variance between samples was the ratio between *cis* and *trans* Hi-C contacts (Table EV2), which also appeared to

vary in the other published datasets examined. Samples with high numbers of *trans* contacts had a lower average intensity and resembled a normal distribution, perhaps suggesting that a portion of the *trans* contacts are actually biological noise in the form of randomly generated Hi-C ligation products. Using the distribution of read counts in the *trans* data, we performed a background subtraction (Average Read Count + 1 Standard Deviation), which was subtracted from all interactions, both *cis* and *trans,* across the entire dataset. Finally, in order to properly account for differences in library depth between samples, we converted individual read counts into reads per billion by dividing the signal for each interaction by the total number of reads (*cis* and *trans*) left after background subtraction, and multiplying by 1 billion. Hi-C matrices were highly similar between HindIII and NcoI replicates used (Appendix Fig S19). Results from the NcoI libraries are represented in the main text and Figures. All heatmaps display log-transformed Hi-C interaction data scaled relative to the highest and lowest interactions within that specific heatmap, with the exception of Figure 2H and Appendix Figs S2 and S3, where clipping was done at 80% and 99%, respectively, to emphasize long-range contacts. The heatmaps in Figure 2H and Appendix Figs S2 and S3 were additionally downscaled by a factor of 2 and 4, respectively, using the aggregate function of the R raster package.

Identification of TADs

To define the fundamental Topologically Associating Domains (TADs) we exploit the Directionality Index (DI) measurement introduced originally by (Dixon et al, 2012). From the Hi-C dataset, we measured DI at each bin by calculating the difference in read counts between upstream and downstream interactions within a window of size L. As previously identified, the DI signal becomes roughly independent of the window size for $L > 2$Mb, so in the following analysis we set $L = 2$Mb. We then examined the distribution of DI values (Appendix Fig S4) and their standard deviation, σ. We found the average of DI values is practically zero. We set a relevance threshold, α, and identify the boundaries of TADs as the points along the DNA sequence where DI rises to be above the given threshold ($DI(i) > \alpha\sigma$, left boundary) and where DI declines to be below the threshold ($DI(i) < -\alpha\sigma$, right boundary) (Appendix Fig S4). For any considered value of α (see Supplementary Analyses), the TAD size values are much larger than the TAD sizes calculated in a control random Hi-C matrix obtained by bootstrapping the original Hi-C matrix along its diagonals, which preserves the genomic distance bias. Thus, the boundaries

identified in the case α=0 are significant and, unless otherwise stated, we considered them in our study. Small changes in the threshold values do not alter our results (Appendix Fig S4).

Comparison of TAD boundaries

In order to compare the location of TADs between datasets, we examined the location of TAD boundaries throughout the genome. For each boundary location in a given sample (A), we attempted to identify a conserved boundary in the second sample (B) within 100kb upstream and downstream (Appendix Fig S6). Overlaps are reported as the percentage of boundaries in sample A that have a corresponding boundary in sample B. As the number of boundaries can vary between samples, the two possible directions of comparison result in different percentages, resulting in a slightly asymmetrical overlap matrix. For simplicity, conservation values from both directions were averaged within the main text and Figures. Overall, we see that the locations of TAD boundaries in the NPC sample are similar to those in both ESC and Neurons, while ESC and Neurons share fewer boundaries (Fig 3F). A comparison between HMM-TADs and ESC-J1 TADs derived using our modified approach indicate that the two approaches generally identify overlapping boundaries, with our approach also defining some structures within HMM-TADs (Appendix Fig S6).

TAD conservation

To compare TADs defined by our approach (see above) with the HMM-based approach (HMM-TADs) used previously (Dixon et al, 2012), we used the following procedure. The boundaries of TADs identified with either method in the published ESC-J1 Hi-C data were considered to coincide if their location was within a given tolerance from each other. The tolerance, Δx, used here is 40% of the average TAD length (Δx = 200kb). In cases where both TAD boundaries coincide within the allowed Δx, the two TADs are counted as overlapping. We also used our TAD definition algorithm to define TADs based on ESC-J1. To compare TADs defined by the two different approaches we employed two measures of global overlap: (i) the fraction of overlapping TADs (Appendix Fig S20), a measure previously used (Dixon et al, 2012), and (ii) the fraction of genome within overlapping TADs. The largest global overlap was found for the value of α where the average size of TADs with the two methods is about the same, specifically α around 0.2. In that case the overlap is about 64% by use of measure (i) and 60% by measure (ii)

(Appendix Fig S20). For $\alpha=0$, we found that the overlap is approximately 47% by use of measure (i) and 29% by use of measure (ii). Importantly, in both cases the values found are much higher than the overlap found by considering a randomized system where the same TADs are used but their linear order along the genome was randomized, which is 15% and 9% respectively with measure (i) and (ii). Overall, our results confirm that the fundamental TADs identified with our very restrictive overlapping criterion match those using more elaborate methods (Dixon et al, 2012). The same approach was used to find conserved TADs between different cell types.

Identification of metaTADs

After the identification of the fundamental TADs, we calculate the mean interaction $I_{k,k+1}$ between all the points of neighbouring domain pairs:

$$I_{k,k+1} = \sum_{ij} x_{ij} \,/\, (b_k - a_k)(b_{k+1} - a_{k+1}) \quad (1)$$

where k is the TAD index, x_{ij} are the values of all the entries of the Hi-C matrix corresponding to the two TADs, a_k and b_k (resp. a_{k+1} and b_{k+1}) are the left and right boundary coordinates of TAD k (resp. $k+1$), and the sum i runs from a_k to b_k and j from a_{k+1} to b_{k+1}. If we have n domains, there are $n-1$ neighbouring TADs pairs, and also $n-1$ values for I. Then, we considered the pair having the highest interaction I_{max}, which we grouped together to define what we call a metaTAD, encompassing both TADs. The newly defined metaTAD is added back to the list of domains (whereas its composing subdomains were taken out). The procedure is repeated iteratively until there remains only one metaTAD having the size of the entire chromosome and containing a hierarchy of all prior metaTADs. The coordinates of metaTADs identified here based on the NcoI dataset are available in Table EV3.

Inner and outer interactions of metaTADs

The interaction between metaTADs is defined using equation (1). We identified the mean interaction between the two domains producing a new metaTAD containing n fundamental TADs, and we consider their average $I(n)$. As control, we computed the same interactions between two neighbouring regions of size equal to the original domains, which were randomly located at any other inter-TAD boundary existing at that level of the three. This corresponds to shift b_k and a_{k+1}

in the sum given in equation (1) into new boundary coordinates $b_{k'}$ and $a_{k'+1}$ for two new neighbouring metaTADs, under the conditions: $b_{k'} - a_{k'} = b_k - a_k$ and $b_{k'+1} - a_{k'+1} = b_{k+1} - a_{k+1}$. Subsequently, we calculated, the average control interaction $I_C(n)$ over metaTADs containing n fundamental TADs.

As a further control, we repeat the whole procedure on the Hi-C matrices randomized as described just above: we defined the corresponding metaTAD tree using the above definitions, then we calculated in the same way the quantities $I(n)$ and $I_C(n)$. In this way, we can compare the significance level of the metaTADs found on the real Hi-C matrices with randomized matrices (Appendix Fig S7A-D).

The inner interaction of a metaTAD was defined by the mean of all Hi-C contact entries contained within its boundaries. Consider a metaTAD, k, encompassing n fundamental TADs. We define the quantity $J_k(n)$:

$$J_k(n) = \sum_{ij} x_{ij} / (b_k - a_k)^2$$

where x_{ij} are the values in the metaTAD corresponding Hi-C matrix, a_k and b_k are its left and right boundary coordinates in the Hi-C matrix, and i and j run from a_k to b_k. This is the average inner interaction of metaTAD k. Then, we calculated the average, $J(n)$, over all the metaTADs containing precisely n TADs. As a background reference level, we considered the quantity $J_C(n)$, which is obtained by repeating the above same procedure in a set of random Hi-C matrices, which are generated, as in the case above, by repeated permutation of bins at the same distance from the diagonal (bootstrapping approach). We choose this kind of randomization since it preserves the genomic distance bias.

We find that in all the time points J/J_C as a function of n remains 20% above the expected random background level up to domains approximately 40Mb wide, i.e., with n=80 (Appendix Fig S7E-H). Note that the value of the background normalized interaction, J/J_C, up to those scales, is comparable to the value for n=1, which corresponds to the original TAD. Thus, metaTAD background corrected intra-domain interactions are comparable to those observed in the lowest level TADs, at least for metaTADs up to roughly 40Mb. This analysis further supports the presence of interactions between TADs, which form a hierarchy of larger metaTADs in a self-similar pattern of ‘domains-within-domains’.

Error bars are computed, for a fixed *n*, by considering the distributions over all chromosomes (excluding Y), propagating the error on the ratio. As we consider more than two orders of magnitude in *n*, we use a logarithmic scale and apply a logarithmic binning for $n > 10$.

MetaTADs in different human Hi-C data

To assess if metaTAD hierarchies can also be found in other organisms, we have analysed human IMR90 and ESC-H1 Hi-C data from (Dixon et al, 2012) building the metaTAD trees starting from our fundamental TADs identified with α=0.0. The average TAD size is d_0 = 0.44Mb in hESC-H1 and d_0 = 0.55Mb in IMR90 cells. As the human Hi-C matrices lack the data corresponding to the centromeric regions of the chromosomes, we took that into account by considering separately the two chromosome arms. Accordingly, the random matrices used for the control curves are obtained by excluding the centromeres in the bootstrapping procedure (see section on TAD identification).

Analyzing the resulting metaTAD trees, we found a significant hierarchy of domain contacts by both measures we considered before: interactions between metaTAD pairs are significantly stronger than background interactions both in the case of the I/I_c, ratio (inter-domain interactions, Appendix Fig S8A-C) and in the case of the J/J_c ratio (intra-domain interactions, Appendix Fig S8D-F), up to domains of tens of megabases.

Investigating metaTAD hierarchies on Hi-C interaction matrices corrected for linear distance

In this study, we have used the ICE methods (Imakaev et al, 2012) and background subtraction to normalize the Hi-C matrices, as in most Hi-C studies. An additional option is to consider normalization of interactions for linear distance (see e.g. (Yaffe & Tanay, 2011)). To test the robustness of our results against such proximity effects, we have repeated our metaTAD analysis using a Hi-C matrix corrected for 1D proximity (observed/expected matrix as described in (Lieberman-Aiden et al, 2009)). We compared the sequence of (meta)TAD joining events based on an uncorrected and 1D-proximity corrected matrix and found consistent joins in 72% of cases when allowing a 20% tolerance due to noise in the data. Hence, our metaTAD hierarchy is stable

even in the case of a 1D corrected Hi-C matrices and reflects true preferential interactions between TADs and metaTADs.

Genomic and epigenomic datasets used for correlation and visualization

A variety of genome-wide features were examined in the context of the metaTAD topology. GC content, gene coverage, average exon count and average gene length of individual TADs and 50kb windows were calculated by custom scripts based on sequence and gene annotation data downloaded for mm9 from the UCSC Genome Browser (Karolchik et al, 2014). The Hi-C principal component analysis (PCA) eigenvectors were determined at a resolution of 100kb using a previously published approach (Imakaev et al, 2012; Lieberman-Aiden et al, 2009). To derive one value per TAD, we calculated the average PCA score of the respective regions.

ChIP-seq datasets for RNAPII-S5p (GSM850467), RNAPII-S7p (GSM850468), RNAPII-S2p (GSM850470) as well as H3K36me3 ChIP-seq data (GSM850472) were taken from (Brookes et al, 2012). H3K4me3 (GSM307618), H3K9me3 (GSM307621), H3K27me3 (GSM307619) and H4K20me3 (GSM307622) ChIP-seq data were taken from (Chen et al, 2008; Mikkelsen et al, 2007). H3K27ac ChIP-seq data (GSM970221) was taken from (Ferrari et al, 2014). All ChIP-seq datasets were mapped to mouse build mm9 using bowtie2 v2.0.5 (Langmead & Salzberg, 2012) with default parameters. After mapping, highly duplicated reads (>95% quantile) were removed. Replicates from each experiment were merged. ChIP-seq datasets were normalized by calculating FPKM values from reads falling into each TAD or 50kb window and normalized by the length of the respective region per kilobase and the total number of mapped reads per experiment in millions. Subsequently, we computed log ratios of these signal FPKMs against FPKMs derived from background controls of each individual study. Control for the RNAPII and H3K36me3 datasets was derived from mock ChIP (GSM850473) performed in the absence of antibody (beads only), while controls for H3K4me3, H3K9me3, H3K27me3 and H4K20me3 were ChIPs performed using an antibody against H3 (GSM307624) and the control for H3K27ac was input (GSM970219).

The DNAseI-seq data from ENCODE/University of Washington was downloaded from the UCSC Genome Browser (Encode Project Consortium, 2011) (http://hgdownload-test.cse.ucsc.edu/goldenPath/mm9/encodeDCC/wgEncodeUwDnase/). DNAseI-seq intensity was

calculated as the fraction of each TAD or 50kb window covered by 'DNAseI hotspots' as defined in the UCSC Genome Browser.

Lamina association and replication timing data were obtained from Peric-Hupkes *et al.* (Peric-Hupkes et al, 2010) and Hiratani *et al.* (Hiratani et al, 2008), respectively, as provided for mouse build mm8 from the ReplicationDomain database (Weddington et al, 2008) (http://www.replicationdomain.com). Conversion from mm8 to mm9 coordinates was performed using the *liftOver* tool downloaded from the UCSC Genome Browser. To derive one value per TAD or 50kb window, we calculated the average of all values falling into the respective regions.

To assess transcription factors binding, we downloaded annotated transcription factors peaks for CTCF, Nanog, Oct4, Sox2, Smad1, E2f1, Tcfcp2l1 Zfx, Stat3, Klf4, Esrrb, c-Myc and n-Myc from Chen et al. (Chen et al, 2008). Conversion from mm8 to mm9 coordinates was performed using the *liftOver* tool downloaded from the UCSC Genome Browser. The "All TF" dataset was derived by the union between peaks from any of these datasets, while the "Pluripotency TFs" dataset consists of peaks from Nanog, Oct4, Sox2, Klf4 and Esrrb only. We computed the enrichment of TFs per TAD by counting the number of peaks falling into each normalized by the respective TAD size in Mb.

Processing of CAGE data

CAGE single molecule sequencing datasets were generated for ESC, NPC and Neurons (day 14) and clustered by the FANTOM Consortium (Forrest et al, 2014) and annotated using Ensembl (http://www.ensembl.org/). In the main text, we refer to individual CAGE clusters as transcripts. Expression changes were measured for all CAGE clusters as log2 fold-change between time points, and FDR (Benjamini, 1995) adjusted p-values for differential expression were calculated using DESeq (Anders & Huber, 2010) with default settings. Three transitions in expression were measured: ESC to NPC, NPC to Neurons, and ESC to Neurons; this identified 1117 (793 with Ensembl gene annotation), 38 (31) and 1288 (1093) differentially expressed (adjusted $p <= 0.1$) clusters, respectively. Functional annotation enrichment for clusters was assessed using DAVID (Dennis et al, 2003) and GOrilla (Eden et al, 2009) (Table EV1).

To derive overall CAGE expression measures for TADs or 50kb regions, we calculated FPKM values as the total read count of all CAGE clusters within a TAD or 50kb window normalized by the length of the respective regions in kilobases and the total number of all reads

within the respective CAGE clusters in millions. Finally, FPKM values were log transformed after adding a pseudocount of 1×10^{-7}.

Correlations of biological features over metaTAD trees

To investigate the correlation of biological features with the tree structure, we first defined a distance, $d(i, j)$, on the tree space between two generic TADs i and j. We considered the hierarchical distance, given by the total height of the smallest branch including i and j, as well as the number of edges minus one along the tree path from TAD i to TAD j. Such distances take into account the hierarchical structure of domains within domains. As they both return very similar results, we used the latter (number of edges along the metaTAD tree minus one) throughout the manuscript.

Once we have the tree distances between all the TADs pairs, we collected the subset U_d of the pairs having the same distance d. We then calculated the Pearson correlation coefficient over that set in the biological feature of interest:

$$C(d) = \sum_i (s_{1i} - \mathrm{E}[s_1])(s_{2i} - \mathrm{E}[s_2]) /(\mathrm{Var}[s_1]\ \mathrm{Var}[s_2])^{1/2}$$

where the sum is over the N_d TAD pairs in the set U_d, s_{1i} and s_{2i} are the epigenomic signals of the first and second TADs in the pair number i, $\mathrm{E}[s_1]$ and $\mathrm{E}[s_2]$ are the mean of s_{1i} and s_{2i}, and finally $\mathrm{Var}[s_1]$ and $\mathrm{Var}[s_2]$ are the variance of s_{1i} and s_{2i}. In this way, we obtain the correlation coefficients for different tree distances, d. Specifically, we calculated the correlation coefficient $C(d)$ for each chromosome and evaluated their average. Error bars in Fig 3B and Appendix Fig S11 represent standard errors over the 20 chromosomes examined in the mouse genome.

For better visualization of $C(d)$ and comparison with random tree structures (see below), we converted the tree distance d into the corresponding average genomic distance s (Appendix Fig S21). This was accomplished using the following relation:

$$s(d) = \sum_i (c_{2i} - c_{1i}) / N_d$$

where c_{1i} and c_{2i} are the genomic coordinates of the midpoint of the first and second TAD of pair i in the set U_d. In the plots of our figures, unless otherwise stated, we show the tree correlation function $C(s)$.

In general, we find that the correlation decreases approximately according to a stretched exponential law (superimposed dashed line over the correlation data):

$$C(s) \sim \exp - (s / s_0)^{\beta}$$

where the best fit parameters s_0 and β depend on the considered biological feature. $C(s)$ decreases from 1 to 0 as s becomes larger and larger. We defined the correlation length, L_0, as the genomic distance where $C(s)$ is 20%.

Appendix Fig S11 shows the tree correlation function $C(s)$ for a variety of epigenomic transcription data in ESC, as well as CAGE data for ESC, NPC and Neurons indicating that correlations along the metaTAD tree extend to much longer scales than along the linear genomic distance. For most features, the real tree correlation function is statistically significant above the random tree (see correlation on random neighbour trees paragraph) correlation function (Fig 3C and Appendix Fig S12).

Correlations of biological features over random neighbour trees

We made the same calculation as above, for comparison, for random neighbour trees. Random neighbour trees were built in the following way: starting from the real linear array of TADs, we randomly join neighbouring TADs pairs within metaTADs irrespective of their Hi-C interactions. In this way, each TAD k has the same 50% probability to be joined to its left neighbouring TAD $k-1$ or to its right neighbouring TAD $k+1$. Once a random metaTAD is produced, it is added back to the list and the procedure is iterated up to the size of the whole chromosome as discussed before. The derived random neighbour trees were found to be highly similar in TAD distance distribution to the observed tree as shown in Appendix Fig S22. We then calculated the tree distances d and the corresponding correlation coefficient as described in the previous section. We consider an ensemble of 30 such random tree structures for each chromosome. To measure significance, we computed one-sided Wilcoxon rank sum tests of difference in median between the real and random neighbour trees.

Analysis of genomic and epigenomic features associated with TAD and metaTAD boundaries

We analyzed enrichments of epigenomic features at TAD and metaTAD boundaries. MetaTADs were selected according to size, and those > 10Mb and < 40Mb were used in boundary analyses. Above 40Mb the interactions between metaTADs are not significantly greater than expected, and below 10Mb an increasingly large proportion of boundaries are shared between TADs and metaTADs (as metaTADs are built originally from TADs). Up to two thirds of TAD boundaries are metaTAD boundaries without any size selection.

The genomic region around each TAD or metaTAD boundary was split into 50kb bins extending 450kb in each direction. ChIP-seq datasets (RNAPII, H3K27me3, CTCF) and DNaseI-seq datasets were processed with MACSv2 to give relative signal over background (from an estimated local model), which was then averaged over all boundaries genome wide. For gene density, CAGE TSS (CTSS) density and LAD boundary density, simple intersection counts were used per bin and averaged over all boundaries.

Nuclear lamina association heatmaps display raw microarray probe intensities averaged into the same 50kb bins described above per boundary. Transitions between high and low lamina association were detected by fitting a linear regression model across the boundary bins and classifying those models with an absolute coefficient >0.05 as crossing a lamina transition. Boundaries were divided into those that coincided with a lamina transition and those that did not, and heatmaps were then sorted by average intensity. To test the significance of the association between boundaries and lamina transitions, we circularly permuted both boundaries and transition points on each chromosome 1000 times, and calculated the proportion of boundaries that crossed lamina transitions using the same linear regression procedure. Empirical p-values were then calculated as the number of permuted results greater than or equal to the observed value.

Comparison of Hi-C trees

The hierarchical organization of Hi-C data can be represented in the form of a dendrogram or tree. Here we gauge similarity between the Hi-C trees of ESC, NPC and Neurons, and between these and random trees, by employing two popular tree comparison methods: the Robinson-Foulds

distance (Robinson & Foulds, 1981) and the cophenetic correlation coefficient (Sokal & Rohlf, 1962). The Robinson-Foulds distance between two trees is commonly described as the removal and addition of splits required to transform one tree into the other (Huson, 2011) and can be normalized by 2*(n-3), where n is the number of leaves in the tree. The cophenetic correlation coefficient is the Pearson-correlation between the cophenetic matrices of the trees (distances through the tree between all leaves) being compared (MathWorks, 2014). Albeit different, these two approaches correlate with one another to a certain extent (Spearman correlation equal to -0.426). In both methods, we focused solely on tree topology, and all tree branch lengths were made to be equal to 1.

The matrices in Fig 4B and Appendix Fig S15 summarize the tree comparisons for the cophenetic correlation coefficient and the Robinson-Foulds distance. The results indicate that Hi-C-based trees are more similar among each other than they are to random trees or random trees with themselves. These tree comparisons also highlight that the correlations previously found between the Hi-C data matrices are recapitulated when looking at hierarchical tree topologies alone – for us an indicator that the hierarchical organization of Hi-C can be well-captured by the trees.

The comparisons between Hi-C-based trees for ESC, NPC and Neurons use only the set of TADs with conserved boundaries between individual samples, here called cTADs, while unconserved TADs were pruned from the trees. Conserved TADs (cTADs) were defined as in the section “TAD conservation”. The use of cTADs is important since it ensures we refer and compare the same genomic locus across time points (with a 200kb tolerance corresponding to 4 bins in Hi-C matrices).

In general, we observe a broad distribution of similarity values when comparing trees of the same chromosome across different cell types. Fig EV4A shows the cophenetic correlation coefficient per chromosome for all comparisons between cell types. Large deviations from the mean could reflect bigger rearrangements in chromosomal 3D architecture throughout the neuronal differentiation.

Fig 4B and Appendix Fig S15A, B also shows comparisons of real trees and random trees. Each real tree was compared with two types of random tree models: the random neighbour tree model as described above and completely random trees. The latter are fully random trees with the same number of leaves as the original real tree, but with no constraints on which TADs make up

a clade/metaTAD; these are generated by the `rtree` function in the ape R package (Paradis et al, 2004). Each tree was compared with 100 random trees and the mean similarity was calculated. For the case of comparing random neighbour trees with themselves, 10 were selected from ESC, NPC and Neurons and these were compared with 10 other random neighbour trees.

TAD classification according to gene expression change

Measuring gene expression changes per TAD is not well established, and here we have pioneered the use of double criteria to robustly classify meaningful TAD expression changes over a time point transition. We employ thresholds for both the minimum absolute expression difference (at least a quarter of the inter-quantile range; a third and a sixth were also considered, see Fig EV4C and Appendix Fig S17) and the minimum absolute fold change (at least 1.5x in CAGE FPKM values per TAD; 1.25x and 2x minimum fold-change was also considered, Fig EV4C and Appendix Fig S17).

We applied this method to all TADs with conserved boundaries (cTADs), as defined in the previous section. cTADs showing expression changes exceeding these thresholds can be visualized in Fig EV4C for all time point transitions. Genomic regions of gene expression change (turquoise intervals of Fig 4C) were defined based on cTADs start coordinates.

Integrating local tree changes, expression changes and A/B compartment membership

In order to relate tree structure changes/ conservation with matched gene expression data we devised a local tree change measure for each cTAD (TAD with conserved boundaries) reflecting the structural tree changes around it during a particular time point transition. For a given cTAD, we order all other cTADs in the chromosome according to increasing tree distance over the metaTAD tree of time point A. Neighbour cTADs are defined as all cTADs with distance less or equal than the third cTAD with highest distance. The tree distances of neighbour cTADs in time point A are then compared with the respective distances (for the same cTADs) over the tree of time point B using a root-mean-squared deviation approach such that

$$\text{Tree change } (TAD\ a) = \sqrt{\frac{\sum_{t=1}^{n}(x_{A,t}-x_{B,t})^2}{n}},$$

where A and B refer to the metaTAD trees of different time-points, $x_{A,t}$ and $x_{B,t}$ to the tree distance between cTAD a and neighbouring cTAD t in the metaTAD tree of time-point A and B respectively, and n the total number of neighbouring TADs considered.

To define regions of tree conservation and tree changes we calculated the z-score of the tree change measure over each chromosome. If a cTAD has a z-score<0, it is considered to be within a conserved region and if z-score>0 a region subject to tree structure changes. Analogous to gene expression changes, genomic regions of tree conservation/change were defined by extending the start of a cTAD to the start of the next cTAD and hence may also include regions where TAD boundaries were not conserved. Fig 4A and Appendix Fig S16 include an example of ESC-NPC local tree changes for chr6.

The distribution of local tree changes for all cTADs between ESC-NPC and NPC-Neurons is significantly different (p-value 5.4e-07; Wilcoxon test) and, for a given threshold value of change, ESC-Neurons is the time point transition with more changing cTADs followed by ESC-NPC and NPC-Neurons (Fig EV4B). Additionally, the total genomic distance occupied by tree changes is higher in ESC-NPC than in NPC-Neurons (Fig EV4B).

The fraction of a given TAD covered by B compartment and the fraction covered by lamina-associated domains (Peric-Hupkes et al, 2010) are highly correlated genome-wide as expected (Appendix Fig S16; ESC-NPC chr6 as example). Since A/B compartments have been shown to relate to active/repressed chromatin, we reasoned that regions of tree changes/conservation should be further divided according to A/B membership. A cTAD is said to be in compartment A (or B) if at least 75% of its length (coverage) is in A (or B). If in a time point transition, a cTAD changes its coverage of a given compartment by more than 25% it is classified as an A/B compartment change region. Similar to our findings with regions subject to tree changes, the total genomic distance occupied by A/B compartment changes is higher in ESC-NPC than in NPC-Neurons (Fig EV4B).

The overlaps between cTADs showing expression changes, regions showing altered tree structure, and regions of defined A/B membership were calculated as the intersection of the relevant intervals divided by their union (i.e. the Jaccard Index). The total genomic distance encompassed by each of the types of regions considered when evaluating the overlap with gene expression changes is represented as percentage of genome length in Appendix Fig S17A. We calculate the significance of the Jaccard Index value using 10,000 circular permutations based on

a randomly chosen cTAD start position and report the empirical p-values (Fig 4E-F). As an additional control, we also show the overlaps obtained with regions displaying no significant changes in gene expression (Fig 4F and Appendix Fig S17B).

Expression analysis of conserved tree topologies

We probed the nature of conserved tree topologies between differentiation time points by relating pairs of cTADs (TADs with conserved boundaries) that keep the same tree distance in both trees with their changes in gene expression.

As before, we classified cTADs as ‘up-regulated’ (Up) or ‘down-regulated’ (Down), according to their change in CAGE per-TAD FPKM using the double criteria previously described. A cTAD was called Unchanged (“Unch”), if its fold-change is less than 1.5x and the absolute expression difference is small (lower than a quarter of their inter-quantile range). Appendix Fig S23 summarizes this classification genome-wide for all time point transitions. We also note that this cTADs classification often follows the expression changes of genes within them, for instance, cTAD containing *Nanog* is down-regulated and the cTAD containing midbrain-specific *Th* is up-regulated.

We next found, for each chromosome, pairs of cTADs connected at distance *d* in both trees for each comparison (ESC-Neurons, ESC-NPC, NPC-Neurons,). Considering each distance separately, we then computed the probability of finding a gene-expression coherent cTAD-cTAD pair, i.e., pairs of cTADs which have identical CAGE change classification: [Up-Up], [Unch-Unch] and [Down-Down], normalized by the total number of pairs at that distance. We contrasted this probability with an independent model where the probabilities of a coherent pair were derived from individual class frequencies at distance *d*; a 90% binomial confidence interval (CI) was added to judge significance.

Fig 4G shows that up to a tree distance of 5, higher spatial proximity through differentiation primes cTADs for similar gene expression changes. Due to pruning of non-conserved TADs, a tree distance of 5 in the tree of cTADs corresponds approximately to distance 6-8 in the tree of all TADs - approximately 2-6Mb in genomic distance.

The Strings & Binders Switch polymer model and its parameters

We employed the Strings & Binders Switch (SBS) model (Barbieri et al, 2012; Nicodemi & Prisco, 2009) to model chromatin, represented as a self-avoiding polymer chain on a cubic lattice with lattice linear spacing d_0. In the present case the chain was made of N = 192 beads. In the SBS model, equally spaced, specific beads of the chain are sites of attachment for floating factors (binders). Red (R) beads are attachment sites only for red binding factors, and analogously for green (G) and blue (B) beads. All other beads are inert, as they have no interactions apart from excluded volume effects (and chain length integrity constraints). The chain total length also includes 12 inert beads inserted at each end of the polymer in order to avoid finite size effects on the chain conformation corresponding to the actual simulated chromatin region.

The model parameters are the affinity E between binding site and binder, and the concentration of binders. In our analysis, we assumed equal values for binding energy and concentrations for the different type of binding sites. The data presented in Fig 5 and Appendix Fig S24 have $E_R = E_G = E_B = 4\ k_BT$, values falling in the known range of real transcription factor binding energies (k_B is the Boltzmann constant and T the temperature in Kelvin). For all details see (Barbieri et al, 2012; Nicodemi & Prisco, 2009).

Monte Carlo simulations

The above model was investigated by Metropolis Monte Carlo (MC) computer simulations (Binder, 1997; Binder & Heermann, 2002). Diffusing molecules and polymer beads randomly move from one to a nearest neighbour vertex on the lattice, while single site occupancy and polymer non-breaking constraints are maintained. Binding is only permitted between nearest neighbour particles on the lattice, as in a "lattice gas" model. Our binders have a binding multiplicity of six, close to the typical number of polymerases in a transcription factory. MC averages are over up to 10^4 runs, each run being fully equilibrated with up to 10^{13} single MC steps (Binder, 1997; Binder & Heermann, 2002) (Appendix Fig S24).

Monte Carlo dynamics is suited for sampling the equilibrium conformation ensemble. To estimate also the order of magnitude of the time scales involved in the real polymer dynamics, we estimated the conversion factor from MC steps to real time by matching the diffusion coefficient estimated for DNA loci in mammalian cells ($D \approx 1\text{-}10\mu m^2$ / h) with the one measured in our simulations (Barbieri et al, 2012; Chubb et al, 2002).

Contact matrices, gyration radius, and occupied volume

The contact matrices of the model polymer discussed in the main text report the probability of finding a bead pair within a distance of $3d_0$. The volume occupied by a group of beads, e.g. the red beads, is here taken as the volume of a sphere of radius R_g ($V = 4/3\ \pi\ R_g^3$), where R_g is the gyration radius of those beads, a measure of their average distance from their center of mass.

CryoFISH detection of DNA

ESC (46C) cells were fixed in 4% and 8% paraformaldehyde in 250 mM HEPES (pH 7.6; 10 min and 2h, respectively). Cell pellets were embedded in 2.1 M sucrose in PBS and frozen in liquid nitrogen. Ultrathin cryosections were cut using an EM FC7 ultracryomicrotome (Leica, Wetzlar, Germany) with ~180 nm thickness, captured in sucrose-PBS drops, and transferred to coverslips for cryoFISH.

Cryosections were first washed (2x, 30min total) in 2xSSC, then incubated (2h, 37oC) with 250 µg/ml RNase A (Sigma; in 2xSSC), washed (2x) in 2xSSC permeabilised (10 min) with 0.2% Triton X100 in 2xSSC and washed (3x) in 2xSSC. Cryosections were washed and treated (10 min) with 0.1 M HCl, washed (3x) in PBS and then incubated (15 min) in 20 mM glycine in PBS. Cryosections were then dehydrated in ice-cold ethanol (30%, 50%, 70%, 90% and 3x100%, 3 min each), dried briefly, denatured (10 min, 80°C) in 70% deionized formamide, 2xSSC, 0.05M phosphate buffer pH 7.0, and then re-dehydrated as above. After a brief period of drying, coverslips were overlaid onto probe mixture on Hybrislips (Invitrogen, Eugene, USA) and sealed with rubber cement for *in situ* hybridisation.

Probes consisted of MYtags custom labelled oligonucleotide libraries produced by MYcroarray (Ann Arbor, MI, USA). Regions ‘a’, ‘b’ and ‘c’ were labelled with ATTO-550, ATTO-488 and ATTO- 594.

Probe libraries were precipitated and air-dried and resuspended in deionised 100% formamide. Probe in formamide was mixed 1:1 with a 2x ‘hybridization mixture’ containing 20% dextran sulphate, 0.1 M phosphate buffer (pH 7.0) and 4xSSC. Probes were denatured (10 min) at 70°C before hybridization.

Hybridization was carried out at 37°C in a moist chamber for ~40h. Post-hybridization washes were as follows: 50% formamide in 2xSSC (42°C; 3x over 25 min), 0.1xSSC (60°C, 3x over 20 min), and 0.1% Tween-20 in 4xSSC (42°C, 10 min). Nuclei were counterstained (45 min) with

DAPI in PBS with 0.05% Tween-20, rinsed sequentially in 0.05% Tween-20 in PBS and then PBS alone. Coverslips were mounted in VectaShield (Vector Laboratories, Peterborough, UK) immediately before imaging.

Microscopy and image analyses

Images from cryosections were acquired on a confocal laser-scanning microscope (Leica TCS SP8; 63x objective, NA 1.4) equipped with a 405 nm diode, and a white light laser, using pinhole equivalent to 1 Airy disk. Images from the different channels were collected sequentially to prevent fluorescence bleedthrough. For automated quantitative image analyses, images (TIFF files) were automatically merged and each channel manually thresholded in ImageJ to define masks for nuclei, and for each locus. Distances between the centers of mass of FISH signals within each nucleus were measured using an in-house ImageJ script (kindly provided by Tiago Rito and Miguel Branco). Averages and standard deviations were calculated from 2 independent FISH experiments. Statistical analyses were performed using R. For Figure EV5B, images (TIFF files) were merged in Adobe Photoshop, background noise was removed using the Despeckle filter, before contrast stretching.

Supplementary Analyses

Robustness of TADs identified by different methods in different datasets

The definition of TADs considered in our manuscript is based on the Directionality Index (DI) and a significance threshold, α (see Supplementary Methods). As different thresholds may return different TAD boundaries, we explored how TADs depend on α. We first discuss the TAD average size, d, as a function of α: we found it converges to a constant value, d_0, as α tends toward zero (Appendix Fig S4) and such a limit value is d_0 = 0.48Mb, 0.51Mb, and 0.47Mb in our ESC, NPC, and Neurons Hi-C datasets (Appendix Fig S5A). The corresponding TAD size distribution, P(d), is approximately exponential and its support extends down to 50kb which is the resolution of the Hi-C NcoI dataset considered here (Appendix Fig S5B). For small α values, the size of TADs is only weakly dependent on the threshold. Importantly, their average size is much larger than the TAD sizes computed in a control random Hi-C matrix obtained by bootstrapping the original Hi-C matrix along its diagonals (which preserves the genomic distance bias). Thus, the boundaries identified in the case α = 0 are significant and we considered this case in our study. The coordinates of TADs identified here from the NcoI dataset are available in Table EV3.

To illustrate the effects of the threshold, here we explore the TADs obtained for two values, α=0.0 (used in the main text) and α=0.2, for the identification of TADs in our ESC data; we also consider the case where our method is applied, with a threshold α=0.2, to ESC-J1 data from (Dixon et al, 2012), in order to compare the results with the original TADs identified with the HMM method (Dixon et al, 2012). The distributions of TAD sizes based on different datasets and TAD definition methods are reported in Appendix Fig S25A. The TADs analyzed in the main text (ESC, α=0) have an average length of ~500kb, which places them between the local domains defined by (Rao et al, 2014) (~300kb) and the original TADs defined by (Dixon et al, 2012) (~1Mb). Using a DI threshold of α=0.2 results in TADs with the same size as Dixon *et al.* (~1Mb), both in our and the Dixon's murine ESC Hi-C datasets.

To compare the different methods and datasets, we determined the distribution of the number of our TADs which overlap the TADs from the original HMM-based algorithm and the TADs identified by use of α=0.2 (both in our ESC and Dixon's ESC-J1 dataset). As expected by considering the average size of individual TADs in the different cases, we find that on average 2

TADs from the "α=0 case" overlap just one TAD from the HMM or the "α=0.2 case" (Appendix Fig S25B). However, in 40-50% of cases, we see a one-to-one relation between α=0 TADs and TADs of any other definition. In addition, among the settings with ~1 Mb-sized TADs, almost all resulting TADs overlap exactly one TAD in any other definition.

Robustness of metaTADs identified from different TAD definitions in different murine Hi-C data

To strengthen the conclusions on metaTADs discussed in our main text, we repeated our analysis of metaTADs using different TAD definitions (see previous section) and using an alternative murine dataset (ESC-J1) from (Dixon et al, 2012).

In Appendix Fig S26, we report genome wide averages of I/Ic and J/Jc using TADs defined with α=0.2 in our own Hi-C dataset (Appendix Fig S26C-D), the ESC-J1 dataset (Appendix Fig S26E-F) as well as the original TAD definitions by (Dixon et al, 2012) based on an HMM (Appendix Fig S26G-H). In all cases, our first measure (I/Ic) indicates significantly higher interactions up to metaTADs comprising ~50 TADs. Given the average size of approximately 1Mb in each case (see the paragraph about exploration of TAD size distribution), this corresponds to length scale of ~50Mb. In line with these results, also the second measure (*J/Jc*) reveals curves that are significantly different from random up to genomic distances of tens of megabases. Therefore, we can summarize that our definition and significance of metaTAD hierarchies are independent on the specific methods used to call TADs and on the dataset considered.

Further, we repeated our analysis of a subset of epigenomic feature correlations for TADs defined with α=0.2 in our own Hi-C dataset and compared it with the correlations measured over the linear genomic distance of TADs, as discussed in Main Text and in the paragraph "Correlations of biological features over metaTAD trees" (Appendix Fig S27A). Finally, we also compared correlations of the same epigenomic features over the metaTAD tree and over random neighbour trees generated from the linear sequence of TADs (Appendix Fig S27B). As presented in the main text for metaTADs based on α=0 TADs, there are significant statistical differences between the real and random cases, up to scales of tens of megabases.

Description of Supplementary Tables

Table EV1: Gene ontology enrichment analysis.
Excel spreadsheet containing enrichment of GO process, function and component terms for all genes represented by CAGE clusters ranked by log2 (fold change) between time points.

Table EV2: Hi-C sequencing and processing statistics.
Excel spreadsheet containing information on read counts for all considered samples, as well as the effect of processing on the data at each step.

Table EV3: NcoI TADs and metaTADs.
Excel spreadsheet containing the coordinates of TADs and metaTADs for the NcoI dataset, binned at 50kb intervals.

Supplementary References

Anders S, Huber W (2010) Differential expression analysis for sequence count data. *Genome Biology* **11:** R106

Barbieri M, Chotalia M, Fraser J, Lavitas LM, Dostie J, Pombo A, Nicodemi M (2012) Complexity of chromatin folding is captured by the strings and binders switch model. *Proc Natl Acad Sci U S A* **109:** 16173-16178

Benjamini Y, Hochberg, Y. (1995) Controlling the False Discovery Rate: A Practical and Powerful Approach to Multiple Testing. *Journal of the Royal Statistical Society* **57:** 289-300

Binder K (1997) Applications of Monte Carlo methods to statistical physics. *Reports on Progress in Physics* **60:** 487-559

Binder K, Heermann D (2002) *Monte Carlo Simulation in Statistical Physics*: Springer-Verlag.

Brookes E, de Santiago I, Hebenstreit D, Morris KJ, Carroll T, Xie SQ, Stock JK, Heidemann M, Eick D, Nozaki N, Kimura H, Ragoussis J, Teichmann SA, Pombo A (2012) Polycomb associates genome-wide with a specific RNA polymerase II variant, and regulates metabolic genes in ESCs. *Cell Stem Cell* **10:** 157-170

Chen X, Xu H, Yuan P, Fang F, Huss M, Vega VB, Wong E, Orlov YL, Zhang W, Jiang J, Loh YH, Yeo HC, Yeo ZX, Narang V, Govindarajan KR, Leong B, Shahab A, Ruan Y, Bourque G, Sung WK et al (2008) Integration of external signaling pathways with the core transcriptional network in embryonic stem cells. *Cell* **133:** 1106-1117

Chubb JR, Boyle S, Perry P, Bickmore WA (2002) Chromatin motion is constrained by association with nuclear compartments in human cells. *Current Biology* **12:** 439-445

Dennis G, Jr., Sherman BT, Hosack DA, Yang J, Gao W, Lane HC, Lempicki RA (2003) DAVID: Database for Annotation, Visualization, and Integrated Discovery. *Genome Biol* **4:** P3

Dixon JR, Selvaraj S, Yue F, Kim A, Li Y, Shen Y, Hu M, Liu JS, Ren B (2012) Topological domains in mammalian genomes identified by analysis of chromatin interactions. *Nature* **485:** 376-380

Eden E, Navon R, Steinfeld I, Lipson D, Yakhini Z (2009) GOrilla: a tool for discovery and visualization of enriched GO terms in ranked gene lists. *BMC Bioinformatics* **10:** 48

Encode Project Consortium (2011) A user's guide to the encyclopedia of DNA elements (ENCODE). *PLoS Biol* **9:** e1001046

Ferrari KJ, Scelfo A, Jammula S, Cuomo A, Barozzi I, Stutzer A, Fischle W, Bonaldi T, Pasini D (2014) Polycomb-dependent H3K27me1 and H3K27me2 regulate active transcription and enhancer fidelity. *Molecular cell* **53:** 49-62

Forrest ARR, Kawaji H, Rehli M, Baillie JK, de Hoon MJL, Haberle V, Lassmann T, Kulakovskiy IV, Lizio M, Itoh M, Andersson R, Mungall CJ, Meehan TF, Schmeier S, Bertin N, Jorgensen M, Dimont E, Arner E, Schmidl C, Schaefer U et al (2014) A promoter-level mammalian expression atlas. *Nature* **507:** 462-470

Hiratani I, Ryba T, Itoh M, Yokochi T, Schwaiger M, Chang CW, Lyou Y, Townes TM, Schubeler D, Gilbert DM (2008) Global reorganization of replication domains during embryonic stem cell differentiation. *PLoS Biol* **6:** e245

Hu M, Deng K, Selvaraj S, Qin Z, Ren B, Liu JS (2012) HiCNorm: removing biases in Hi-C data via Poisson regression. *Bioinformatics* **28:** 3131-3133

Huson DH, Rupp, R., and Scornavacca, C. (2011) *Phylogenetic Networks Concepts, Algorithms and Applications*: Cambridge University Press.

Imakaev M, Fudenberg G, McCord RP, Naumova N, Goloborodko A, Lajoie BR, Dekker J, Mirny LA (2012) Iterative correction of Hi-C data reveals hallmarks of chromosome organization. *Nat Methods* **9:** 999-1003

Itoh M, Kojima M, Nagao-Sato S, Saijo E, Lassmann T, Kanamori-Katayama M, Kaiho A, Lizio M, Kawaji H, Carninci P, Forrest AR, Hayashizaki Y (2012) Automated workflow for preparation of cDNA for cap analysis of gene expression on a single molecule sequencer. *PLoS One* **7:** e30809

Jaeger I, Arber C, Risner-Janiczek JR, Kuechler J, Pritzsche D, Chen IC, Naveenan T, Ungless MA, Li M (2011) Temporally controlled modulation of FGF/ERK signaling directs midbrain dopaminergic neural progenitor fate in mouse and human pluripotent stem cells. *Development* **138:** 4363-4374

Kanamori-Katayama M, Itoh M, Kawaji H, Lassmann T, Katayama S, Kojima M, Bertin N, Kaiho A, Ninomiya N, Daub CO, Carninci P, Forrest AR, Hayashizaki Y (2011) Unamplified cap analysis of gene expression on a single-molecule sequencer. *Genome Res* **21:** 1150-1159

Karolchik D, Barber GP, Casper J, Clawson H, Cline MS, Diekhans M, Dreszer TR, Fujita PA, Guruvadoo L, Haeussler M, Harte RA, Heitner S, Hinrichs AS, Learned K, Lee BT, Li CH, Raney BJ, Rhead B, Rosenbloom KR, Sloan CA et al (2014) The UCSC Genome Browser database: 2014 update. *Nucleic Acids Res* **42:** 1-7

Langmead B, Salzberg SL (2012) Fast gapped-read alignment with Bowtie 2. *Nat Methods* **9:** 357-359

Lieberman-Aiden E, van Berkum NL, Williams L, Imakaev M, Ragoczy T, Telling A, Amit I, Lajoie BR, Sabo PJ, Dorschner MO, Sandstrom R, Bernstein B, Bender MA, Groudine M, Gnirke A, Stamatoyannopoulos J, Mirny LA, Lander ES, Dekker J (2009) Comprehensive

mapping of long-range interactions reveals folding principles of the human genome. *Science* **326:** 289-293

MathWorks. (2014) Cophenetic Correlation coefficient. Vol. 2014.

Mikkelsen TS, Ku M, Jaffe DB, Issac B, Lieberman E, Giannoukos G, Alvarez P, Brockman W, Kim TK, Koche RP, Lee W, Mendenhall E, O'Donovan A, Presser A, Russ C, Xie X, Meissner A, Wernig M, Jaenisch R, Nusbaum C et al (2007) Genome-wide maps of chromatin state in pluripotent and lineage-committed cells. *Nature* **448:** 553-560

Nicodemi M, Prisco A (2009) Thermodynamic pathways to genome spatial organization in the cell nucleus. *Biophys J* **96:** 2168-2177

Paradis E, Claude J, Strimmer K (2004) APE: Analyses of Phylogenetics and Evolution in R language. *Bioinformatics* **20:** 289-290

Peric-Hupkes D, Meuleman W, Pagie L, Bruggeman SW, Solovei I, Brugman W, Graf S, Flicek P, Kerkhoven RM, van Lohuizen M, Reinders M, Wessels L, van Steensel B (2010) Molecular maps of the reorganization of genome-nuclear lamina interactions during differentiation. *Molecular cell* **38:** 603-613

Rao SS, Huntley MH, Durand NC, Stamenova EK, Bochkov ID, Robinson JT, Sanborn AL, Machol I, Omer AD, Lander ES, Aiden EL (2014) A 3D map of the human genome at kilobase resolution reveals principles of chromatin looping. *Cell* **159:** 1665-1680

Robinson DF, Foulds LR (1981) Comparison of phylogenetic trees. *Mathematical Biosciences* **53:** 131-147

Sokal RR, Rohlf FJ (1962) The Comparison of Dendrograms by Objective Methods. *Taxon* **11:** 33-40

Weddington N, Stuy A, Hiratani I, Ryba T, Yokochi T, Gilbert DM (2008) ReplicationDomain: a visualization tool and comparative database for genome-wide replication timing data. *BMC Bioinformatics* **9:** 530

Yaffe E, Tanay A (2011) Probabilistic modeling of Hi-C contact maps eliminates systematic biases to characterize global chromosomal architecture. *Nat Genet* **43:** 1059-1065

Ying QL, Stavridis M, Griffiths D, Li M, Smith A (2003) Conversion of embryonic stem cells into neuroectodermal precursors in adherent monoculture. *Nature Biotechnology* **21:** 183-186

Appendix Figures

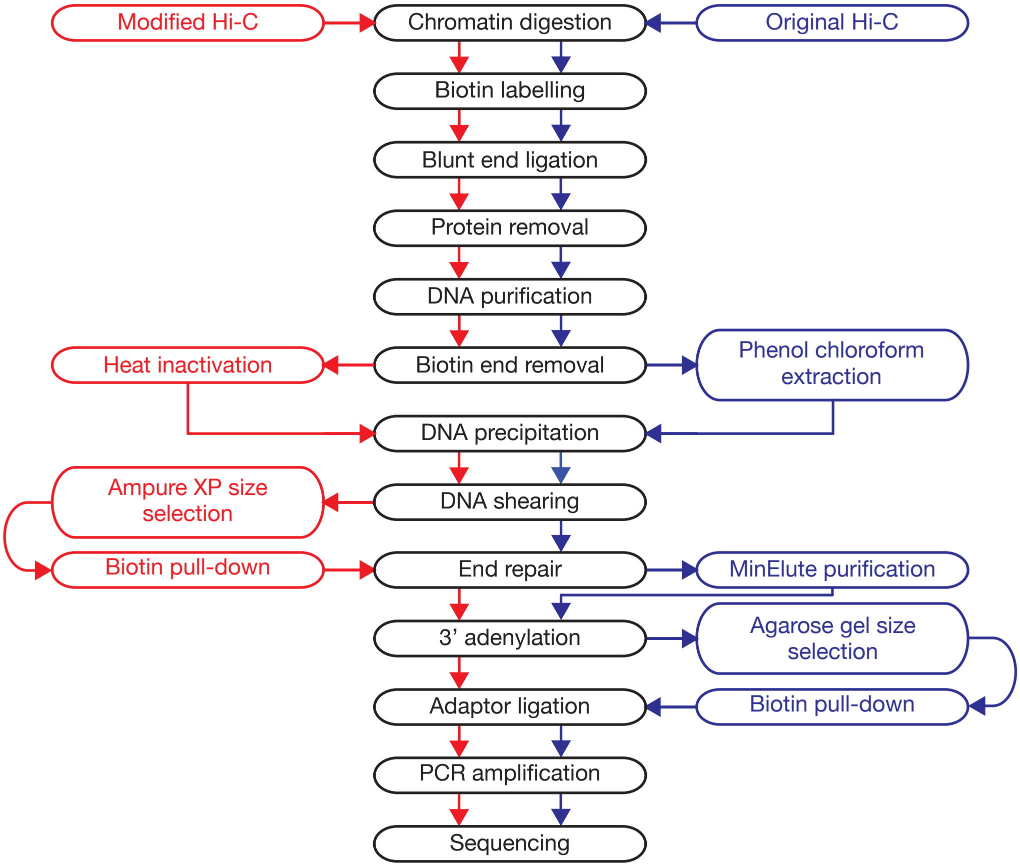

Appendix Figure S1. Comparison of Hi-C protocols.
Overview of the steps in the Hi-C protocol that were adjusted to reduce the number of necessary PCR cycles needed for amplification in order to minimize duplicated reads. The black bubbles in the center represent steps common to the protocol used in this study and the protocol presented in Lieberman-Aiden *et al.* (2009). The order of steps within the original protocol is represented by the blue arrows and bubbles on the right, while the order of the steps in the modified protocol are represented by the red arrows and bubbles on the left. For more details, see Supplementary Information (Extended Methods).

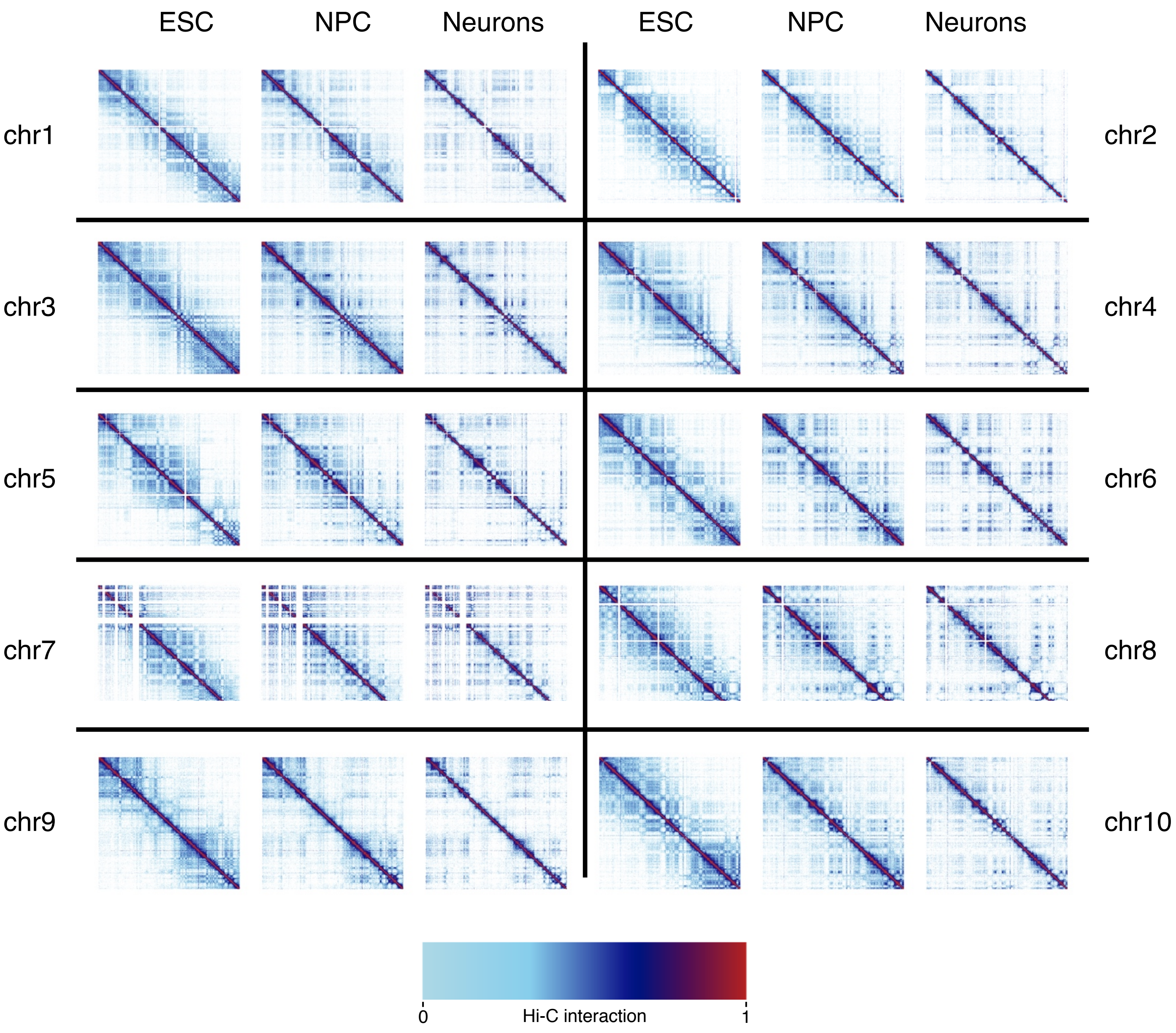

Appendix Figure S2. Changes in long-range interactions for chr1-chr10.
Complex changes in long-range chromatin interactions of many chromosomes are prominent when plotted during the differentiation time series. Hi-C interaction data is plotted in log-scale.

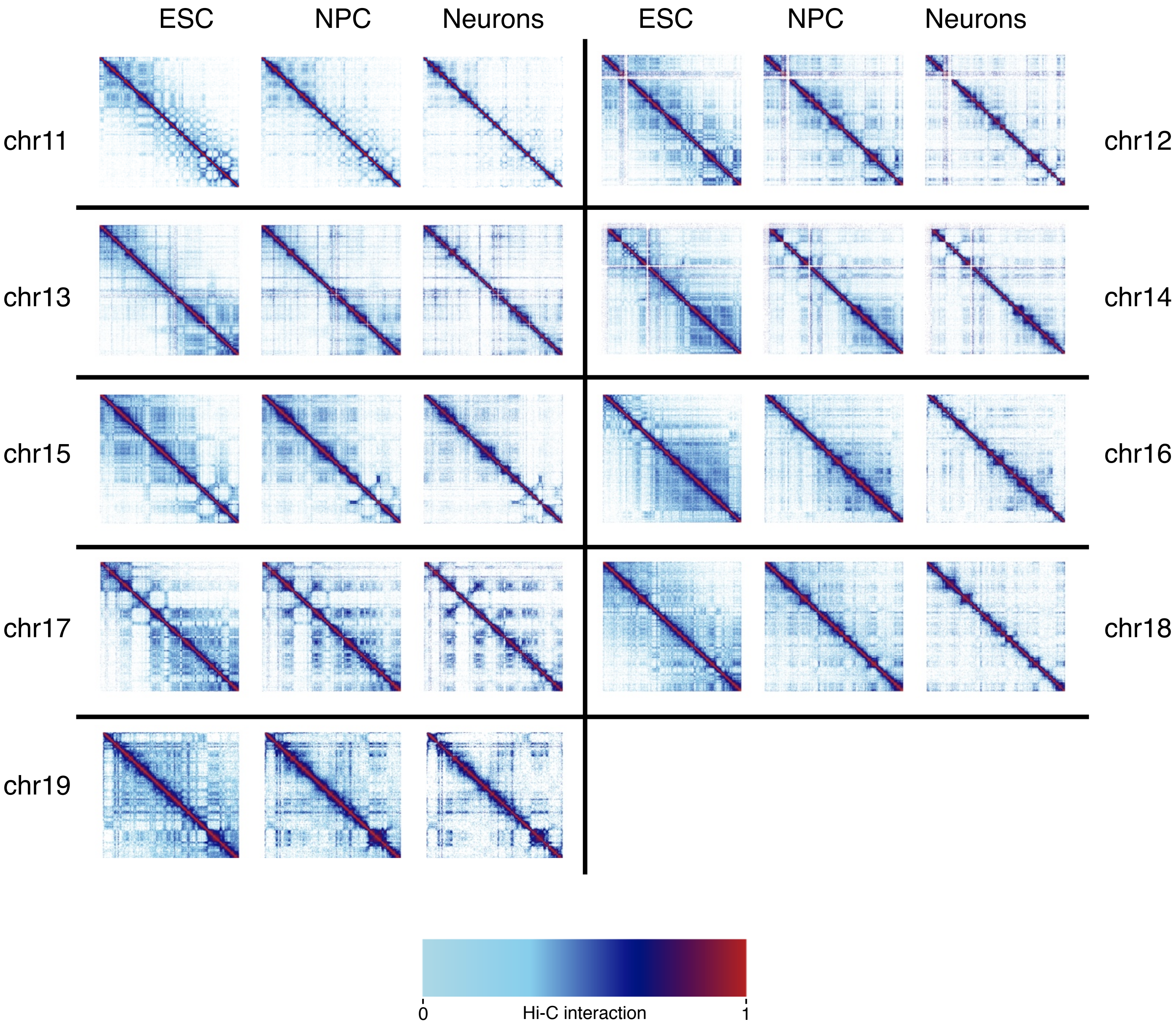

Appendix Figure S3. Changes in long-range interactions for chr11-chr19.
Complex changes in long-range chromatin interactions of many chromosomes are prominent when plotted during the differentiation time series. Hi-C interaction data is plotted in log-scale.

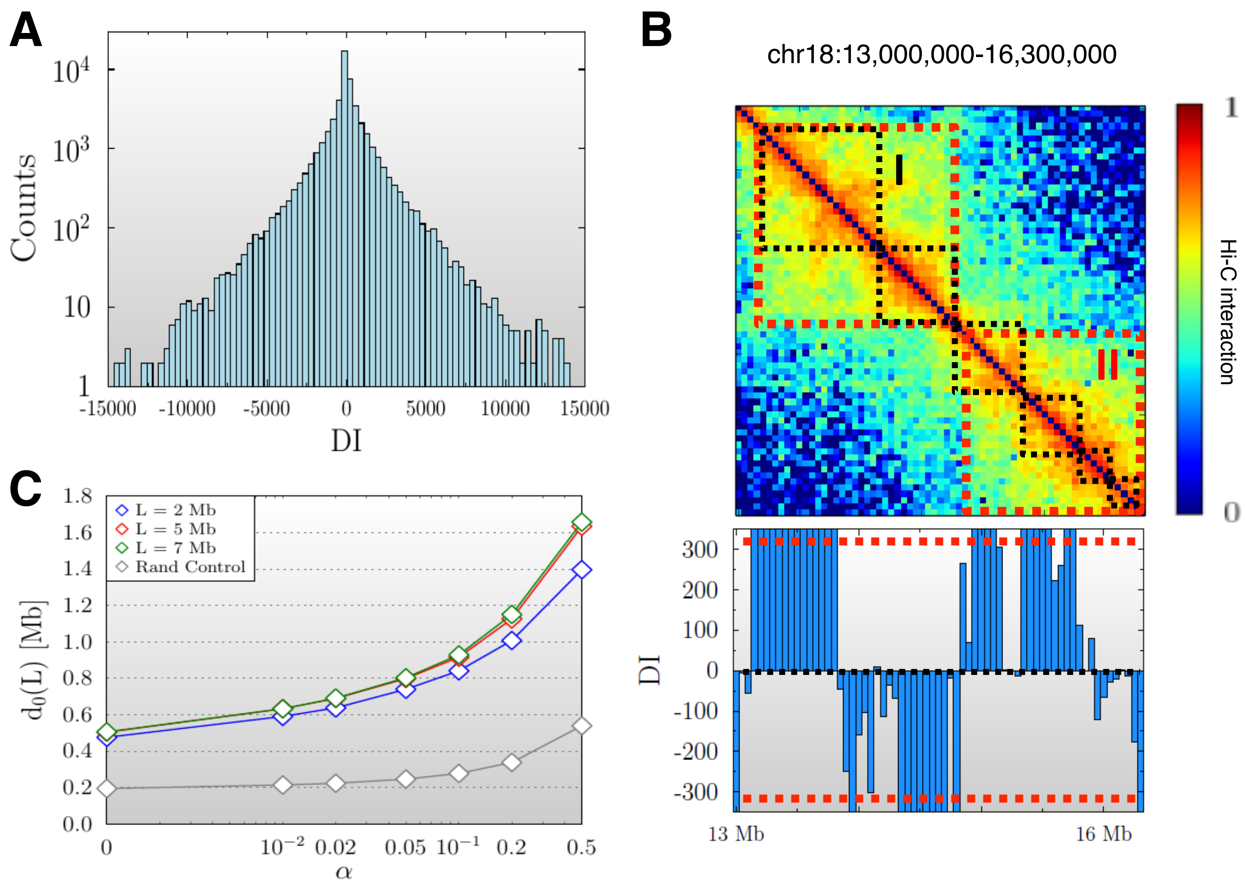

Appendix Figure S4. Identification of TADs.

A Distribution of values of the Directionality Index, DI in the ESC Hi-C data (see Appendix Supplementary Methods, the window size for the calculation of DI is here L=2Mb). The average value of DI is μ = 3, very close to zero, and its standard deviation is σ = 1680. Similar distributions, with analogous values for μ and σ, are found in NPC and Neurons.

B TAD boundaries are identified by positions where |DI| grows above a given threshold ασ, where σ is the standard deviation of DI and α is a threshold constant. Different thresholds return different TADs boundaries: for example, in the Hi-C matrix shown, α = 0 gives the six TADs highlighted by the black dashed lines (TADs **I**), while α = 0.2 gives the two TADs in red (TADs **II**).

C The plot shows the average TAD length d_0 as a function of α, for three values of L, in ESC. The bottom grey line corresponds to the TADs in a control randomized Hi-C matrix (see text). Even for α = 0, the TADs identified in the real Hi-C matrix are well above the random case. d_0 changes only by a factor two, when α increases roughly up to 0.2, while afterwards a much steeper increase is observed. In subsequent analyses, we chose α = 0 to identify TADs.

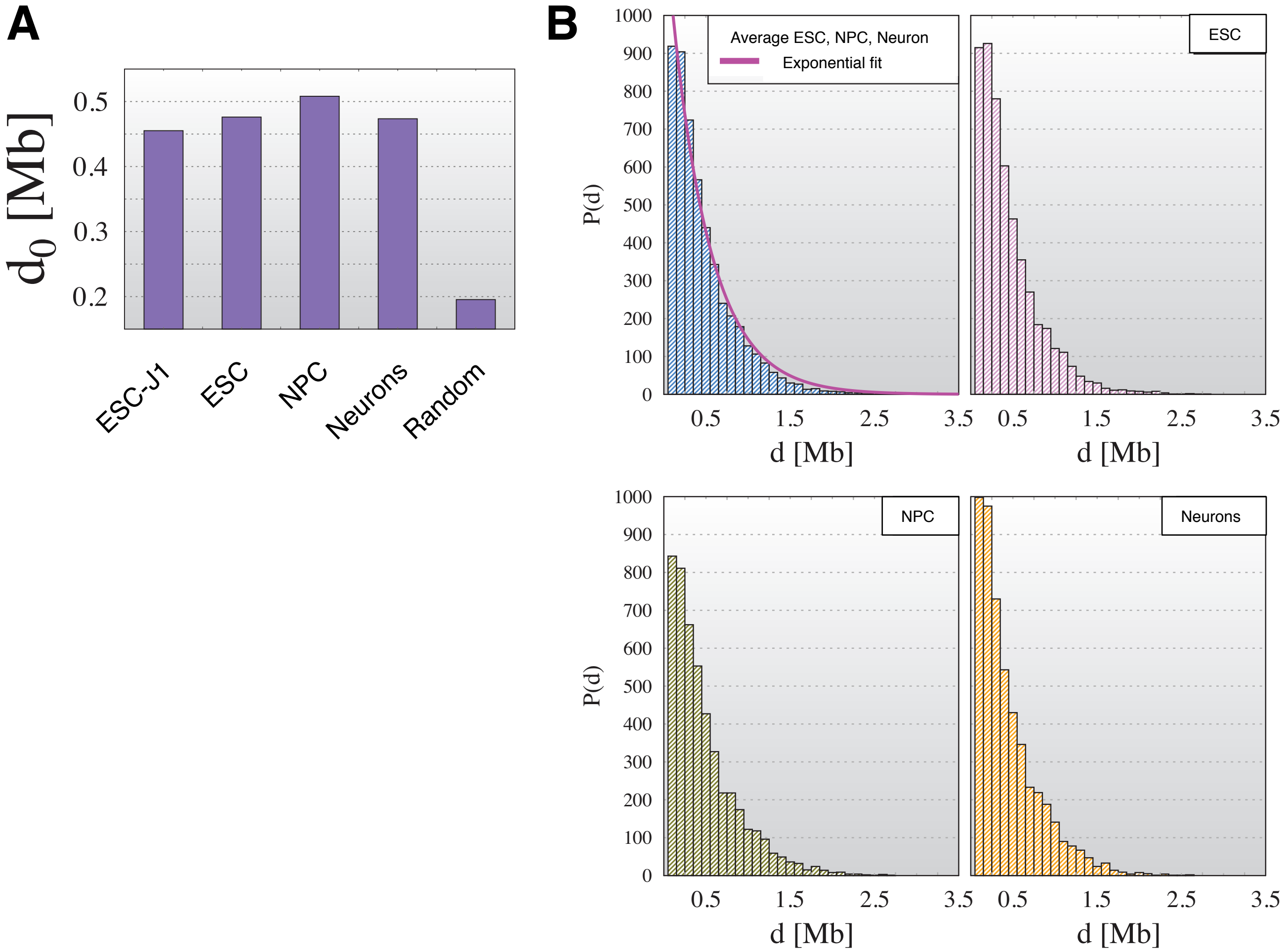

Appendix Figure S5. Size of TADs.

A The average TAD length, d_0 (for α = 0 and L = 2Mb), was measured for the three datasets produced in this study (ESC, NPC, Neurons) and for the ESC-J1 published data (Dixon *et al.*, 2012). The random case corresponds to TADs identified in a randomized Hi-C matrix (see Appendix Supplementary Methods).

B The distribution of TAD sizes is roughly exponential in all our datasets. A detailed comparison of results using different TAD detection approaches is shown and discussed in the Appendix Supplementary Analyses.

Comparing To (B)

Comparing From (A)	# of Boundaries	ESC	NPC	Neurons	ESC-J1	ESC-J1 (HMM)	Random
		5243	4915	5259	5452	3127	3127
ESC	5243	100.00	75.61	74.44	85.89	46.67	26.32
NPC	4915	80.53	100.00	82.64	81.14	47.49	25.94
Neurons	5259	74.44	77.75	100.00	76.67	43.43	26.51
ESC-J1	5452	83.00	73.50	73.73	100.00	45.54	26.45
ESC-J1 (HMM)	3127	89.93	86.02	84.11	92.17	100.00	39.00
Random	3127	44.20	41.00	44.39	46.18	25.36	100.00

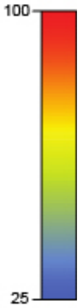

Appendix Figure S6. Overlap of TAD boundaries between datasets.
Here we examine the similarity in location of TAD boundaries between datasets. TADs for ESC, NPC, Neurons and ESC-J1 were identified using $\alpha = 0$. TADs in ESC-J1 (HMM) correspond to the one published in Dixon et al. 2012. The locations of boundaries of the datasets indicated on the left of the table **A** are matched to corresponding boundaries within a 100kb window in the datasets mentioned at the top **B**. Values reported are the percentage of **A** boundaries that have a corresponding boundary in **B**. As the total number of boundaries (specified in the white cells) varies between samples, the table is not symmetrical. This is in contrast to the display used in Figure 3F, where conservation scores are averaged for both comparisons to generate a simpler symmetrical matrix.

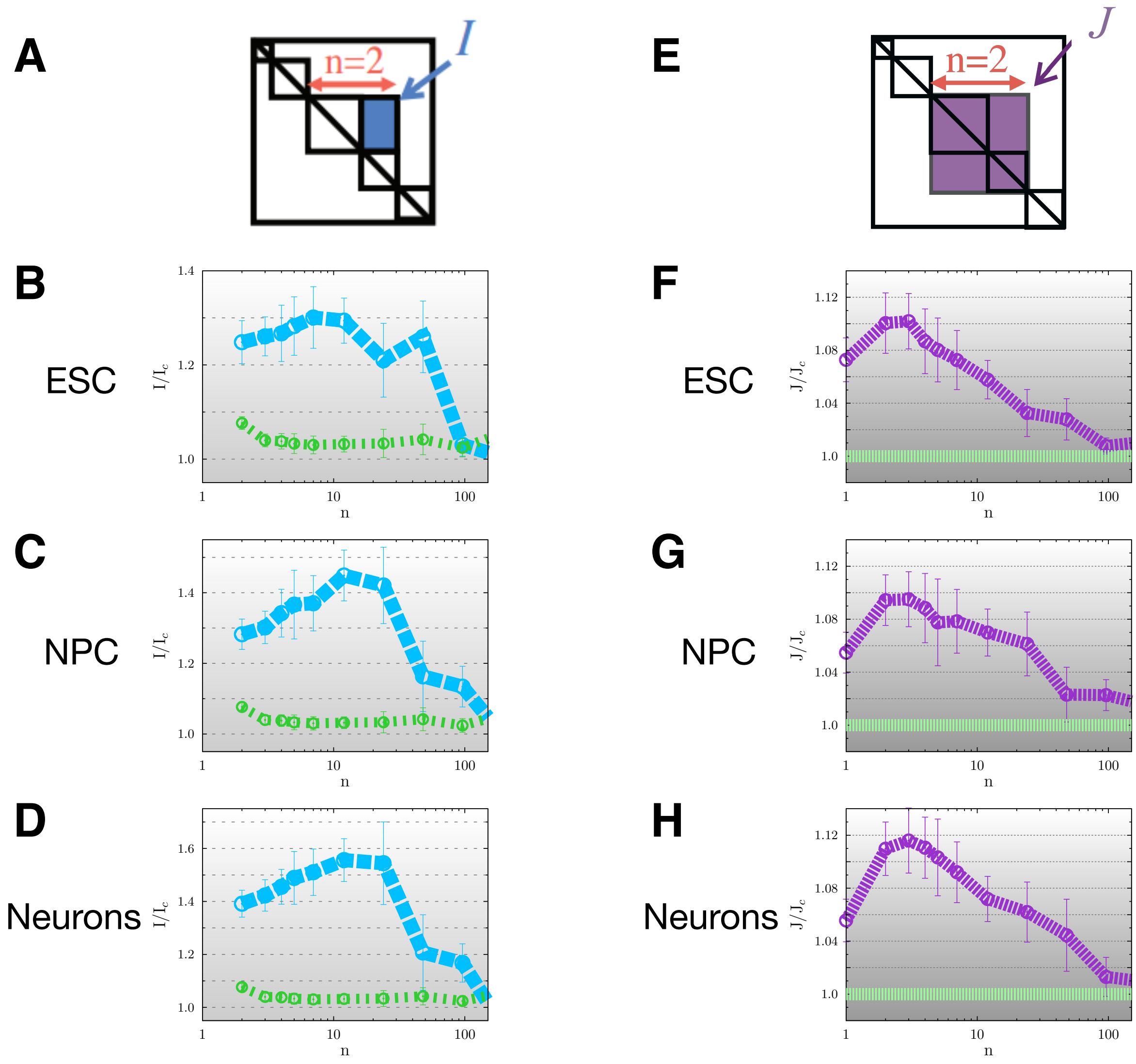

Appendix Figure S7. Measure of inter- and intra-domain interactions.
The background normalized metaTAD inter-domain, I/I_C (panels **A-D**), and intra-domain, J/J_C (panels **E-H**), interactions are shown for our three cell types. The I/I_C ratio is described in the Main Text (see also Appendix Supplementary Methods). J/J_C is the background normalized average contacts within metaTADs. It is shown as a function of the number of TADs, *n*, which a metaTAD includes. J/J_C remains significantly above the control level up to scales of the order of n=40 TADs. The data shown here are the same shown in Main Text Figure 2D, but here we show the corresponding error bars. The enrichment of intra-interactions, J/J_C, in metaTADs is comparable to the enrichment in the original TADs (corresponding to n=1). The value of J_C is the average interaction within a region with the same genomic size as the metaTAD, but calculated on a random Hi-C control matrix generated by repeated permutation of bins at the same distance from the diagonal ("bootstrapping approach"), preserving the genomic distance bias.

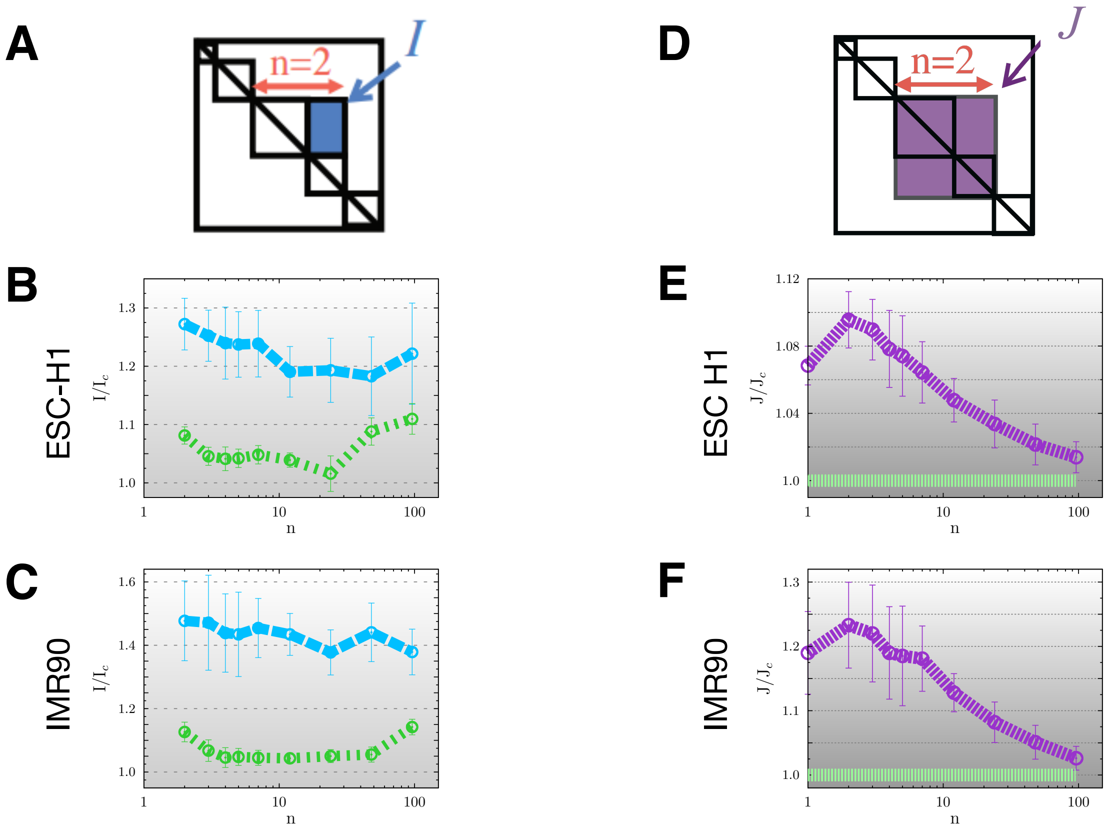

Appendix Figure S8. MetaTAD trees can be found in human cells.
The figure shows the genome averaged I/I_c (blue) and J/J_c (purple) curves, and the corresponding control cases (in green), in different human Hi-C data from Dixon *et al.* (2012). TADs were identified with our detection method using α=0 applied to ESC-H1 (panels **B** and **E**) and IMR90 (panels **C** and **F**) data.

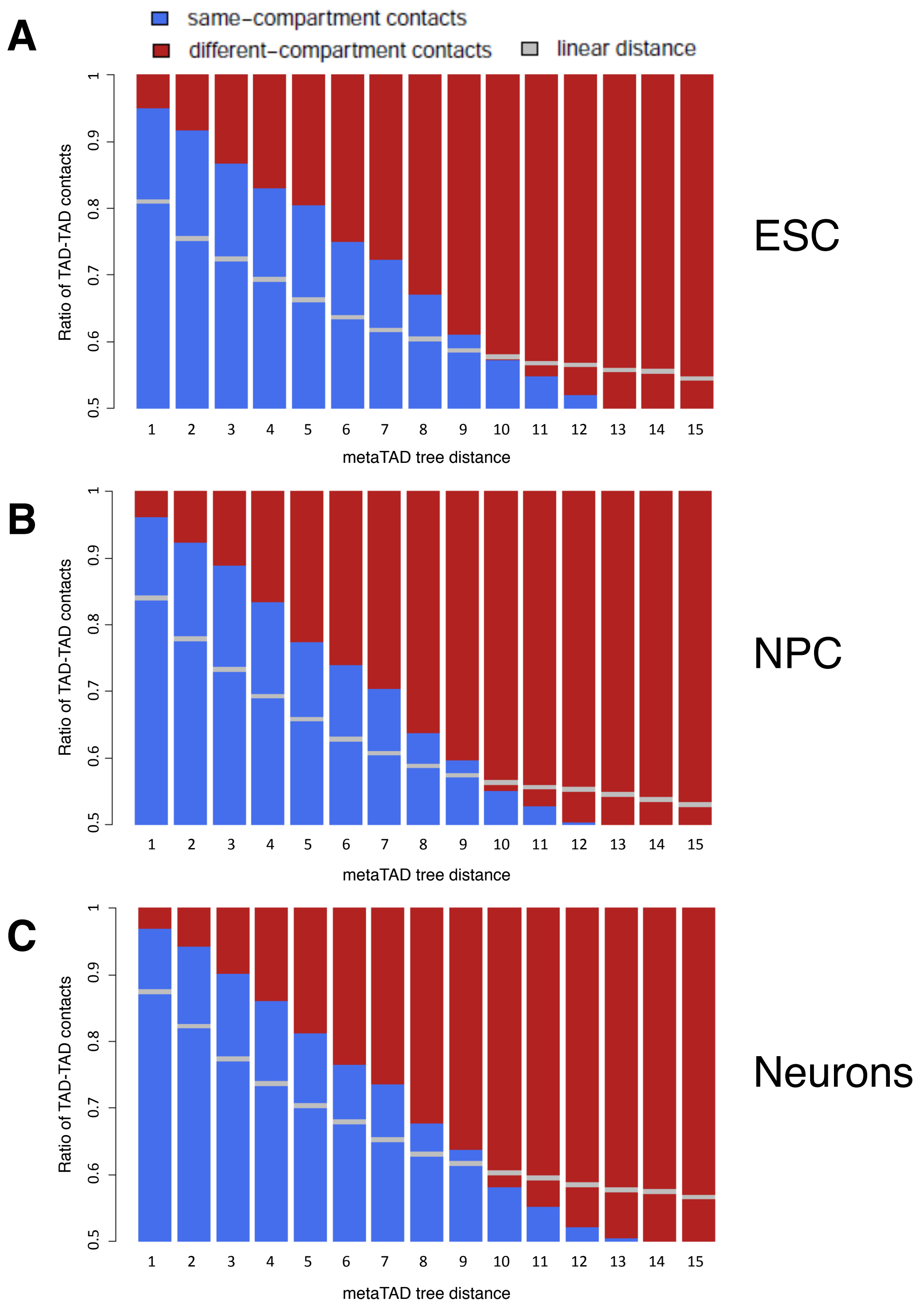

Appendix Figure S9. MetaTAD trees coincide with A/B compartments.
For each distance in the metaTAD tree, we measured the frequency with which two interacting TADs are formed to belong to the same compartment (blue) or different compartments (red). For ESC (**A**), NPC (**B**) and Neurons (**C**), this frequency is much higher than expected from their linear distance (grey) up to metaTAD tree distances of 7 and more.

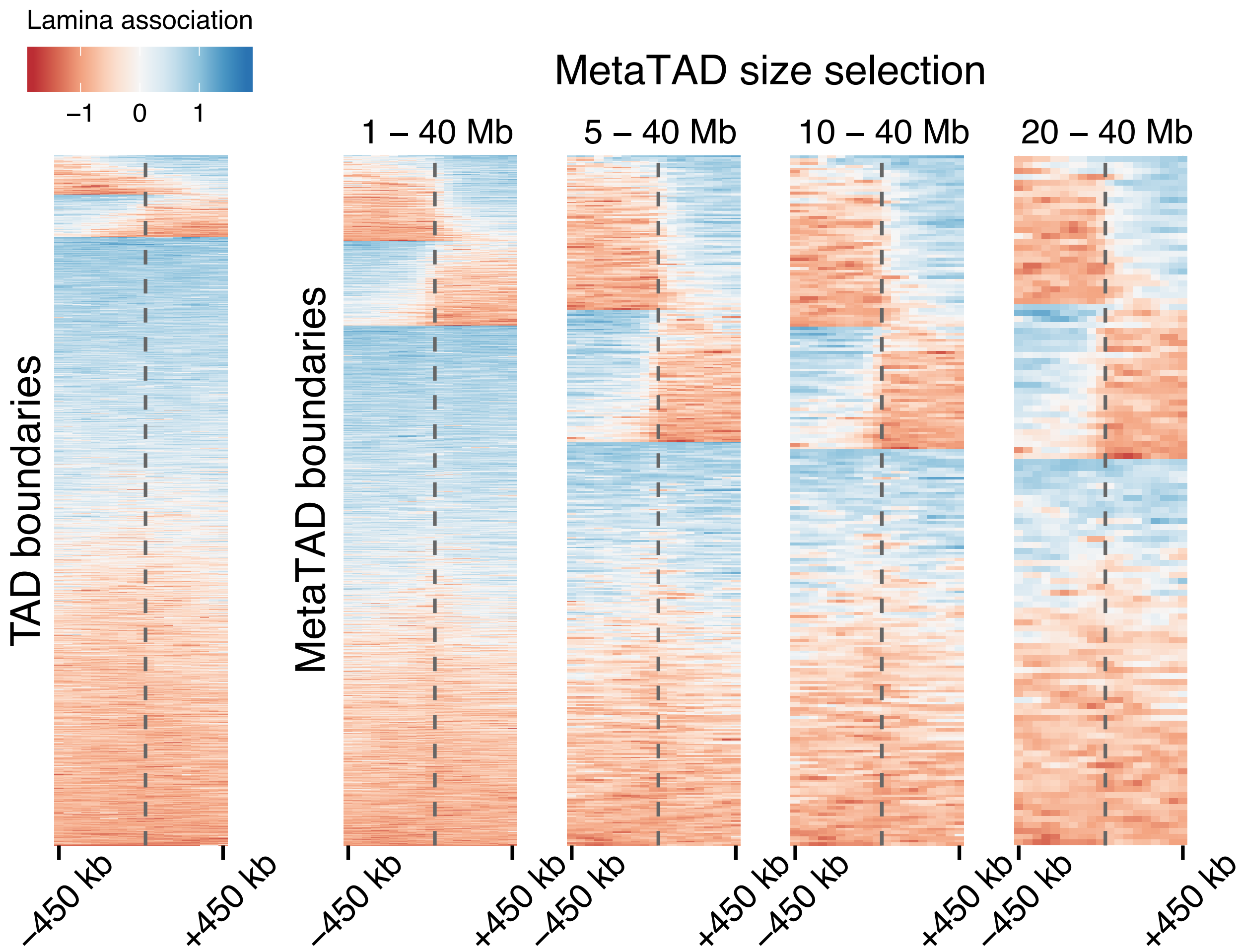

Appendix Figure S10. Transitions of Lamina-association are observed at TAD and metaTAD domain boundaries. Heatmaps depict Lamina-associated (blue) and Lamina-detached (red) regions for a variety of domain boundaries. The leftmost heatmap displays data for all TAD boundaries. The other heatmaps display the data thresholded to select boundaries for metaTAD domains of different lengths. Each heatmap displays the 900 kb flanking domain boundaries (dashed lines) for all boundary regions (heatmap rows). Transitions in lamina association are visible in each heatmap as abrupt changes in colours at boundaries (see Appendix Supplementary Methods) regardless of the size selection thresholds.

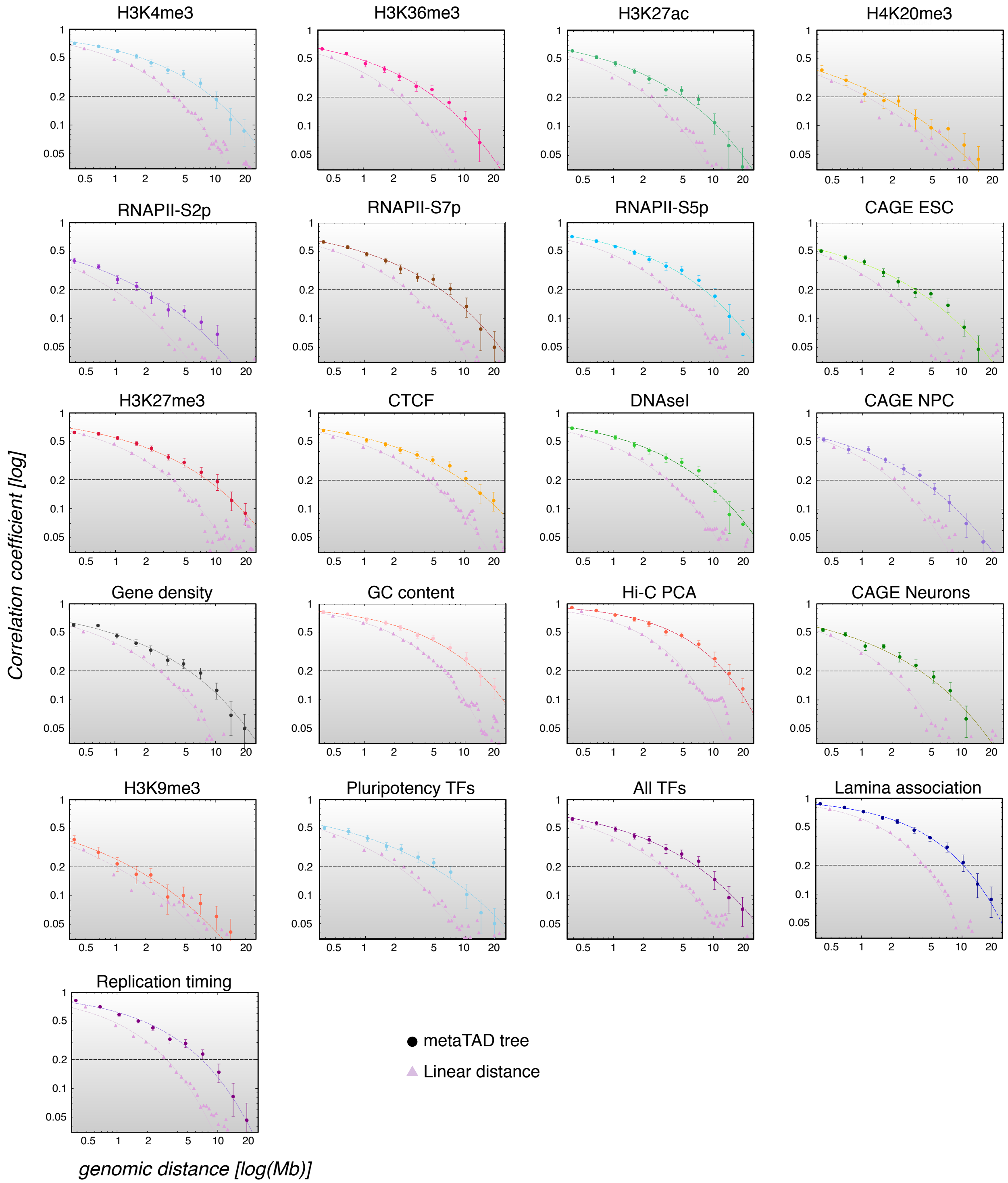

Appendix Figure S11. Correlation of genomic, epigenomic and TF features along metaTAD trees is much stronger than along linear genomic distances.

Pearson correlations of epigenetic and CAGE data, and transcription factor (TF) binding sites over the metaTAD hierarchy (filled circles, upper lines; transformed to average genomic distance; both axes are logarithmic) is much larger than the same correlation measured over the linear genomic distance of TADs (filled triangles, lower lines). See Appendix Supplementary Methods for further details. Superimposed lines are stretched exponential fits.

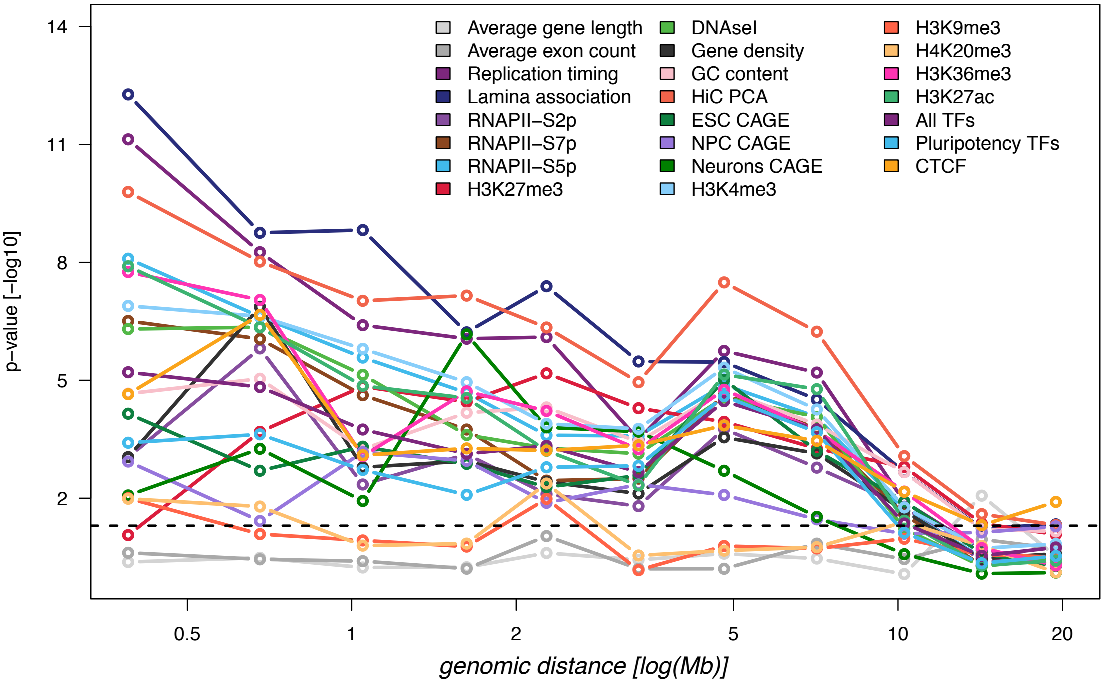

Appendix Figure S12. Correlation of features along the metaTAD hierarchy in comparison with random neighbor trees.

To measure significance, we computed one-sided Wilcoxon tests of difference in median between the real and random neighbor tree correlations (see Appendix Supplementary Methods) and found the former to be significantly stronger up to large distances, approximately 10 Mb, for a number of features; horizontal line, p-value=0.05. Average gene length and exon count per TAD were used as controls.

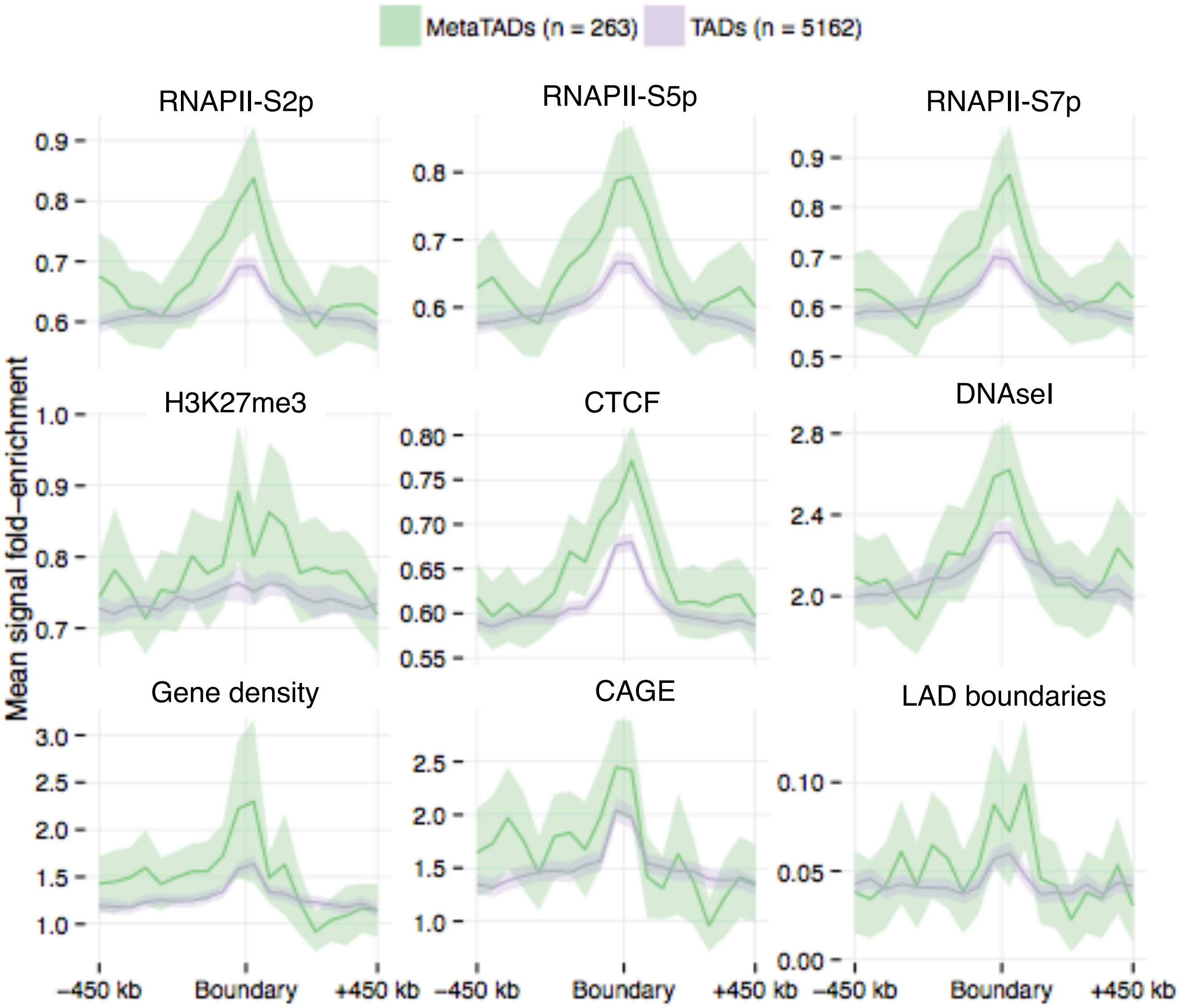

Appendix Figure S13. Comparison of enriched features at TAD and metaTAD boundaries. Genome-wide profiles of epigenomic features and gene densities averaged over all TAD and over only large metaTAD (10 – 40 Mb) boundaries (ribbons show 95% confidence intervals of the mean). The enrichment of most features was significantly increased at metaTAD boundaries when compared to TAD boundaries. 'CAGE' represents CAGE-defined active TSS.

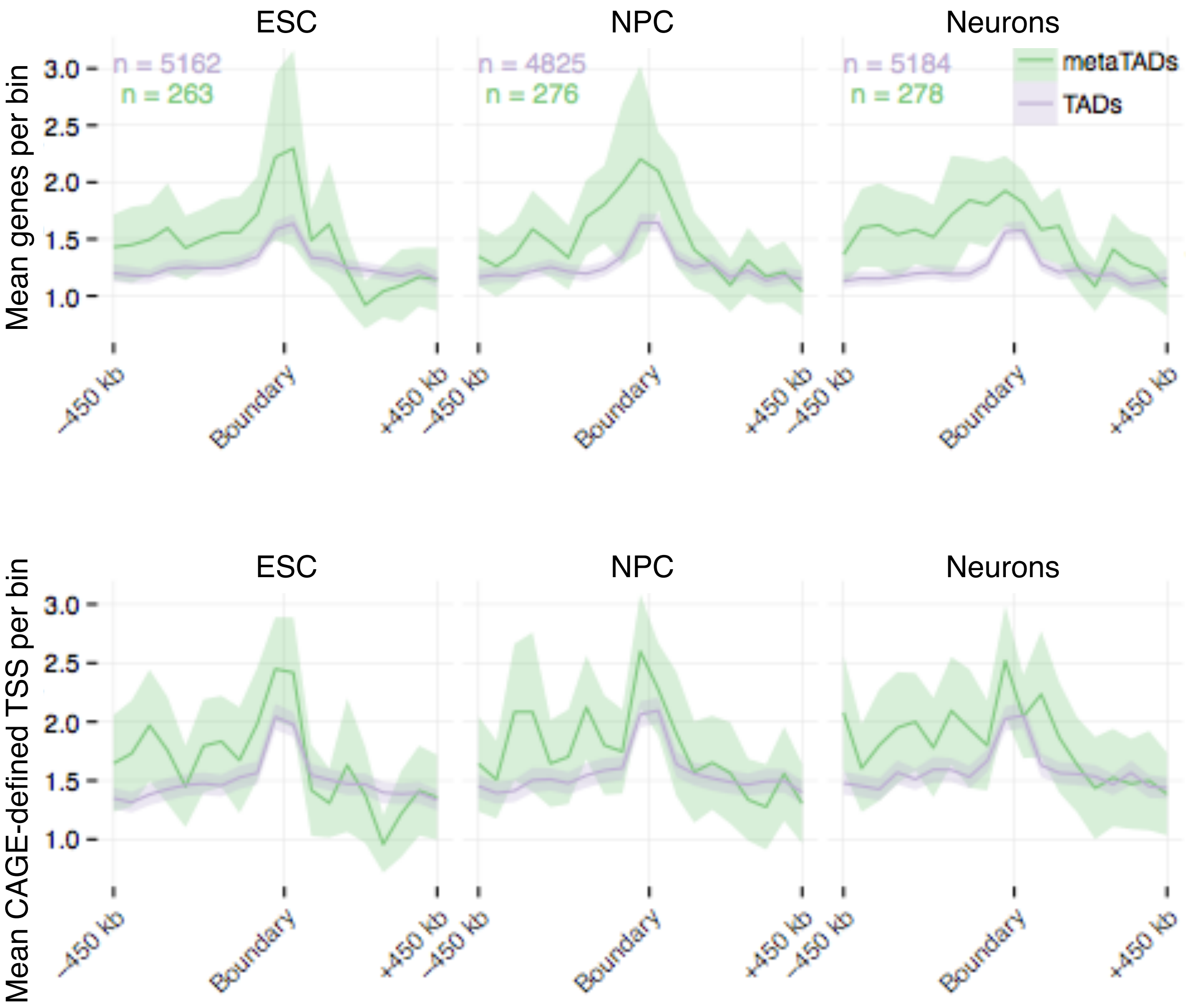

Appendix Figure S14. Consistent gene density and transcribed TSS enrichments (CAGE TSS) at TAD and metaTAD boundaries over the time-course.

Gene densities refer to mean counts of genes per bin, with an overlap of at least 250bp. CAGE-defined active TSS (CAGE TSS) were counted per 50 Kb bin across each TAD and each large metaTAD (10 – 40 Mb) boundary and averaged (ribbons show 95% confidence intervals of the mean). Peak heights suggest modestly stronger enrichments at metaTAD boundaries relative to TAD boundaries**.**

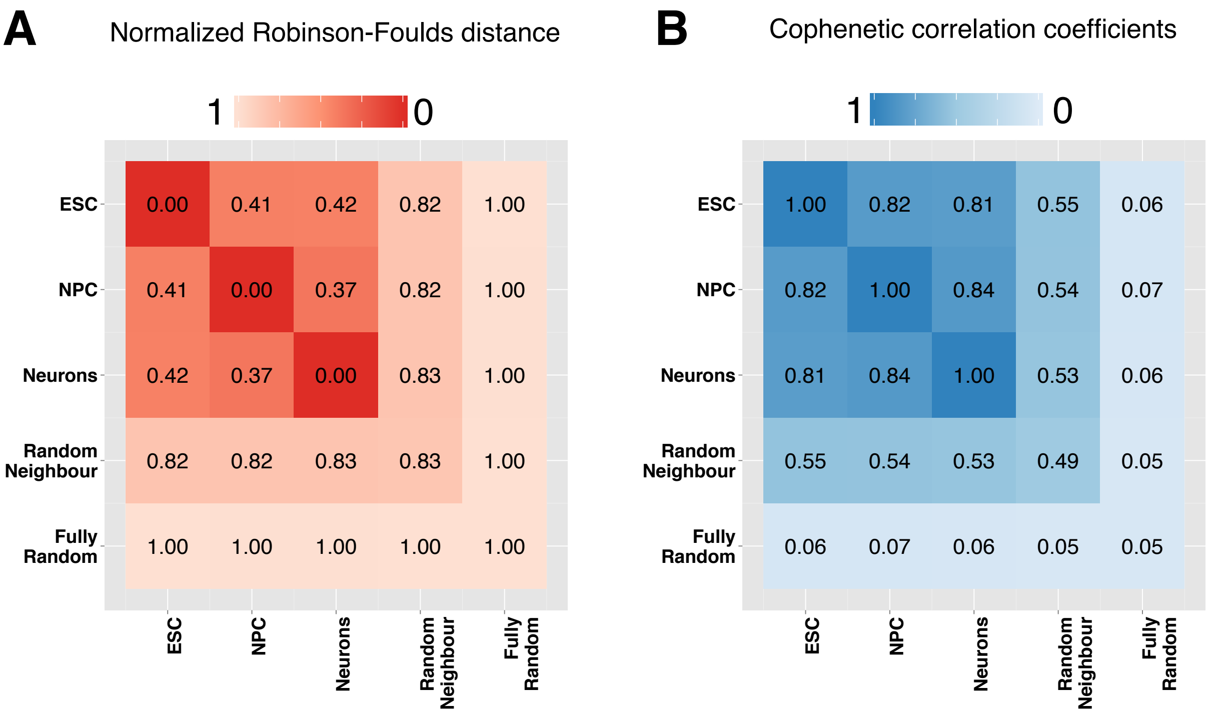

Appendix Figure S15. Comparisons of metaTAD trees between samples.
A,B Normalized Robinson-Foulds distances (**A**) and cophenetic correlation coefficients (**B**; see also Figure 4B) between metaTAD trees composed of TADs with conserved boundaries. Comparisons with two different sets of 100 random tree models are also shown: the random neighbour tree and the fully random tree models (see Appendix Supplementary Methods). Shown are genome-wide averages over all chromosomes. MetaTAD trees are more similar to each other than to random trees. On average, the topology of the NPC metaTAD trees is closer to that of Neurons, both of which are roughly equidistant to the ESC metaTAD trees.

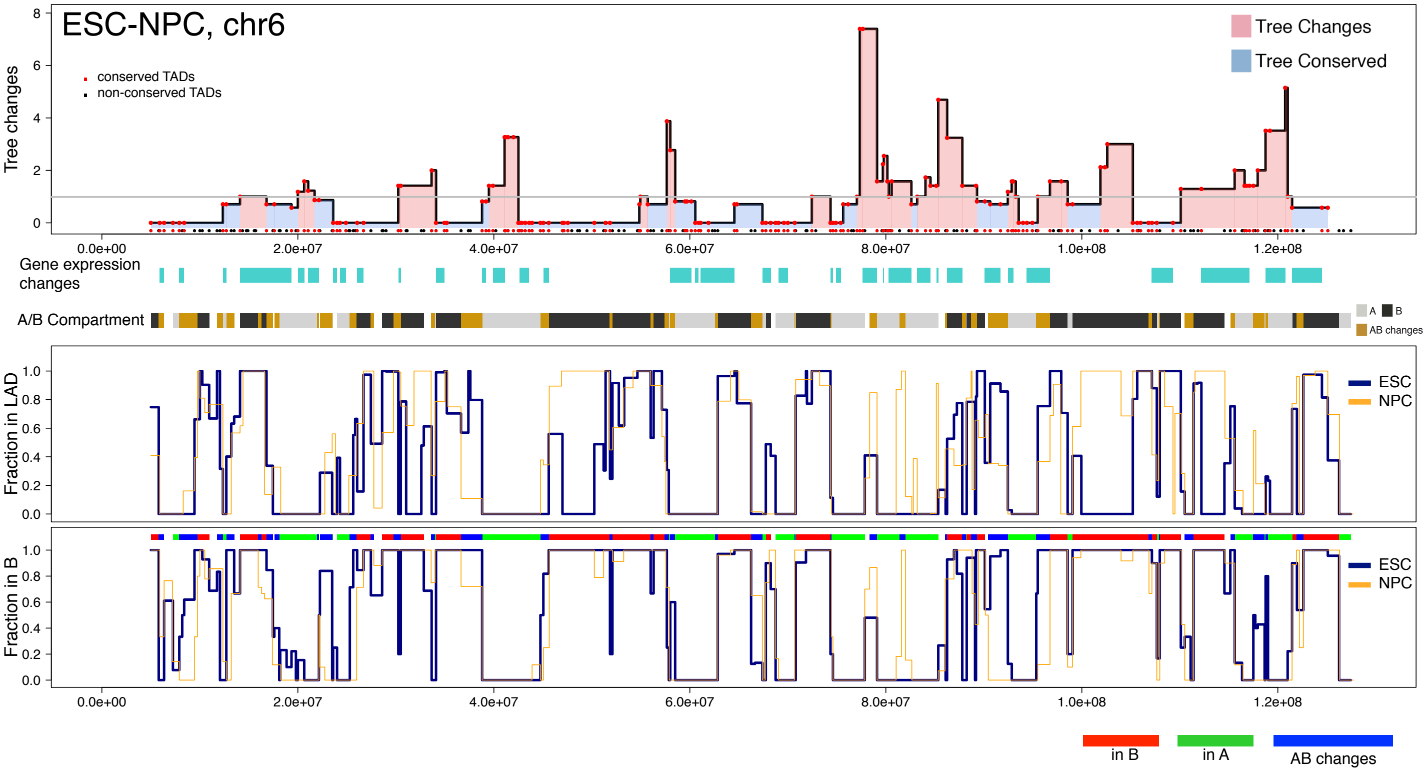

Appendix Figure S16. Comparisons of tree changes with lamina-associated domains and A/B compartments.

Chromosome plot of local tree changes for chr6, ESC-NPC, as depicted in Figure 4A. The grey horizontal line refers to a z-score of zero, used to define genomic regions of tree conservation and tree changes. Dots represent TAD start positions along chromosome. The two lower panels represent the fraction of each TAD covered by a Lamina-associated domain (middle) or by B compartment (bottom panel) for both time-points. If a TAD has over 75% coverage of a given compartment, it was classified as being in that compartment. A TAD with a compartment coverage change of more than 25% was classified as changing.

A

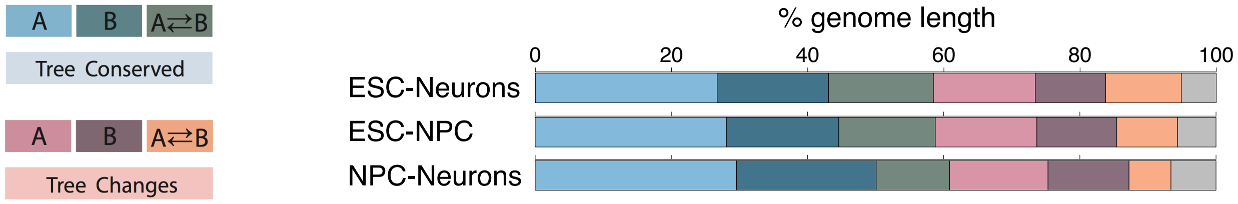

B

Expression changes:

	Fold-change	TAD FPKM difference
A	>1.5x	>IQR/3
in Main Figure - B	>1.5x	>IQR/4
C	>1.5x	>IQR/6
D	>2x	>IQR/3
E	>2x	>IQR/4

	Expression changes criteria	Tree Changes A	Tree Changes B	Tree Changes A⇄B	Tree Conserved A	Tree Conserved B	Tree Conserved A⇄B
ESC - NPC	A	0.12	0.08	0.09	0.17	0.12	0.13
	B	0.12	0.08	0.09	0.17	0.13	0.13
	C	0.11	0.09	0.09	0.16	0.14	0.14
	D	0.10	0.07	0.08	0.13	0.11	0.12
	E	0.10	0.07	0.08	0.12	0.12	0.13
NPC - Neurons	A	0.12	0.07	0.05	0.20	0.15	0.10
	B	0.12	0.08	0.05	0.20	0.17	0.10
	C	0.12	0.08	0.05	0.20	0.18	0.10
	D	0.10	0.06	0.05	0.16	0.14	0.09
	E	0.09	0.07	0.05	0.16	0.15	0.09
ESC - Neurons	A	0.13	0.10	0.12	0.18	0.12	0.13
	B	0.12	0.10	0.12	0.17	0.13	0.13
	C	0.12	0.10	0.12	0.17	0.14	0.13
	D	0.10	0.10	0.11	0.14	0.12	0.12
	E	0.10	0.11	0.12	0.14	0.12	0.12

significance of interval overlap
p-value
0.05
0.01
0.001
<0.0001

No expression changes (control):

	Fold-change	TAD FPKM difference
A	<1.25x	<IQR/6
in Main Figure - B	<1.5x	<IQR/4

	No expression changes criteria	Tree Changes A	Tree Changes B	Tree Changes A⇄B	Tree Conserved A	Tree Conserved B	Tree Conserved A⇄B
ESC - NPC	A	0.03	0.18	0.06	0.04	0.20	0.06
	B	0.04	0.18	0.05	0.06	0.22	0.08
NPC - Neurons	A	0.05	0.14	0.05	0.04	0.21	0.07
	B	0.06	0.16	0.06	0.07	0.21	0.07
ESC - Neurons	A	0.02	0.13	0.06	0.05	0.21	0.09
	B	0.03	0.13	0.06	0.06	0.23	0.10

significance of interval overlap
p-value
0.05
0.01
0.001
<0.0001

Appendix Figure S17. Robustness of the analysis of overlap between gene expression changes and tree changes.

A Genomic proportions of the classified regions used in the overlap analysis.

B Overlap analyses in Figure 4F were repeated for different thresholds classifying TADs with (top) and without (bottom) significant gene expression changes. Empirical p-values were obtained by comparison with 10,000 random circularly permutated intervals.

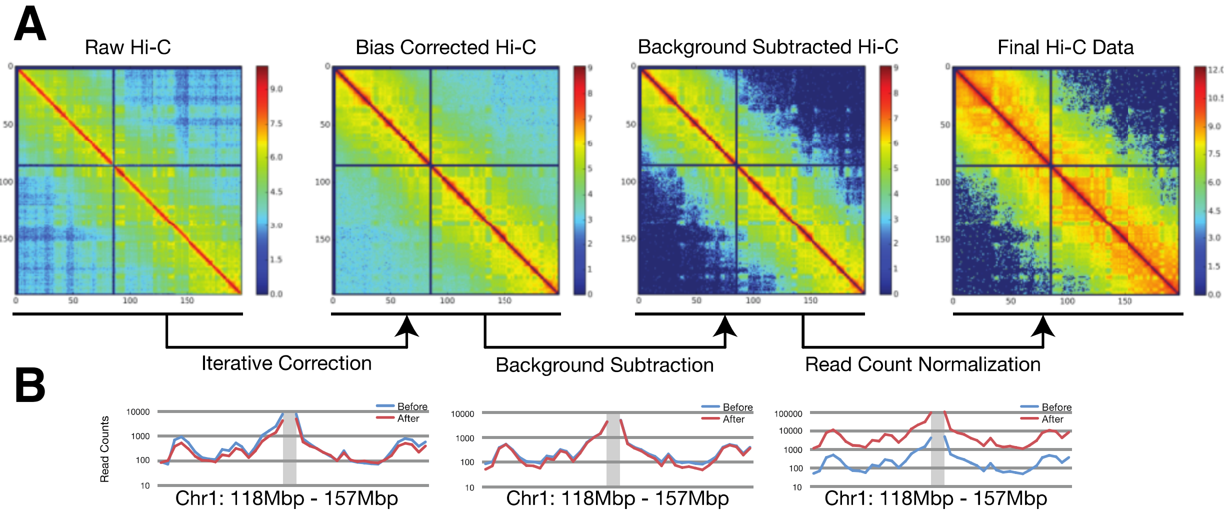

Appendix Figure S18. Hi-C data normalization approach.
(**A**) Hi-C matrices representing chromosome 1 in ESC, and the effects of transformations on the data. The scale is a log representation of the actual values in the matrix scaled automatically to the range of data. (**B**) Visualization of Hi-C interactions before (blue) and after (red) application of each processing steps, as seen 20Mb upstream and downstream from chromosome 1 at 137 Mb (grey column). Data is first corrected using Iterative Correction (Imakaev *et al.*, 2012) to remove experimental biases. Background is calculated using the distribution of *trans* Hi-C reads and is subtracted from both *cis* and *trans* data. Finally, Hi-C data is normalized across datasets by conversion into reads per billion.

		ESC-J1		ESC-46C		NPC		Neurons	
		HindIII	NcoI	HindIII	NcoI	HindIII	NcoI	HindIII	NcoI
ESC-J1	HindIII		0.987	0.946	0.938	0.892	0.886	0.852	0.860
	NcoI	0.987		0.935	0.940	0.879	0.886	0.849	0.857
ESC-46C	HindIII	0.946	0.935		0.941	0.876	0.864	0.820	0.831
	NcoI	0.938	0.940	0.941		0.852	0.891	0.826	0.838
NPC	HindIII	0.892	0.879	0.876	0.852		0.941	0.917	0.921
	NcoI	0.886	0.886	0.864	0.891	0.941		0.918	0.928
Neurons	HindIII	0.852	0.849	0.820	0.826	0.917	0.918		0.970
	NcoI	0.860	0.857	0.831	0.838	0.921	0.928	0.970	

Appendix Figure S19. Spearman correlation of Hi-C data between samples.
Pairwise Spearman correlation coefficients measured between normalized Hi-C datasets binned at 1Mb. Only interactions occurring less than 20Mb apart within each chromosome were considered.

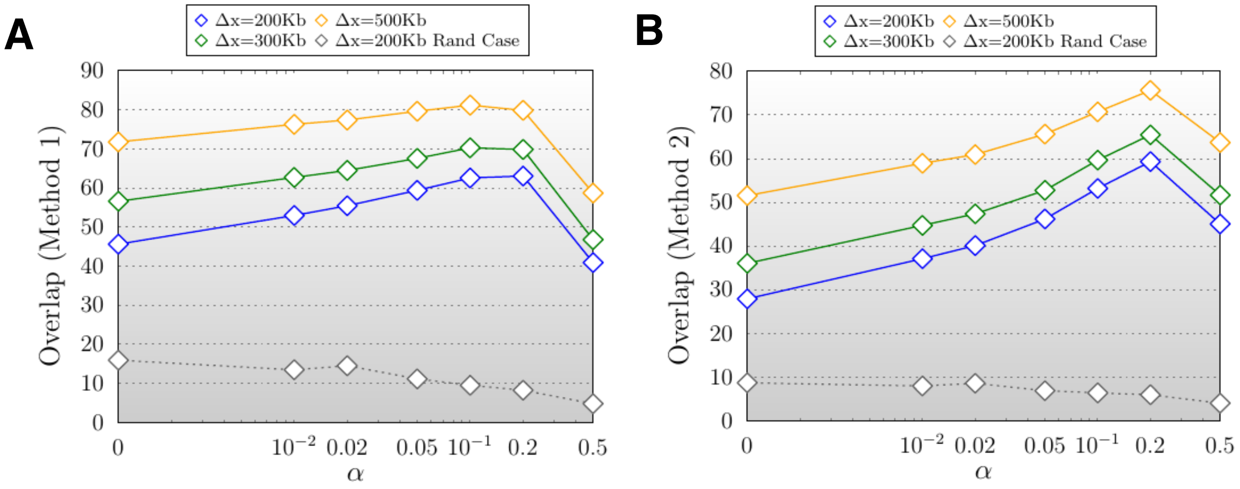

Appendix Figure S20. TAD overlap.
We examine the amount of similarity between HMM-TADs annotated by Dixon *et al.* (2012) and TADs identified in this study based on ESC-J1 cells. The random control used is based on a sample with randomly reshuffled real TADs positions (see Supplementary Methods). (**A**) The overlapping fraction of TADs as defined by Dixon *et al.* (2012) and TADs identified in this study in ESC-J1, as a function of the threshold α for different values of the tolerance, Δx, used to define 'overlap' (see Supplementary Methods). (**B**) A different overlap measure, the fraction of genome within overlapping TADs in our and Dixon's definition. The grey line is the comparison with a random control case (see Supplementary Methods).

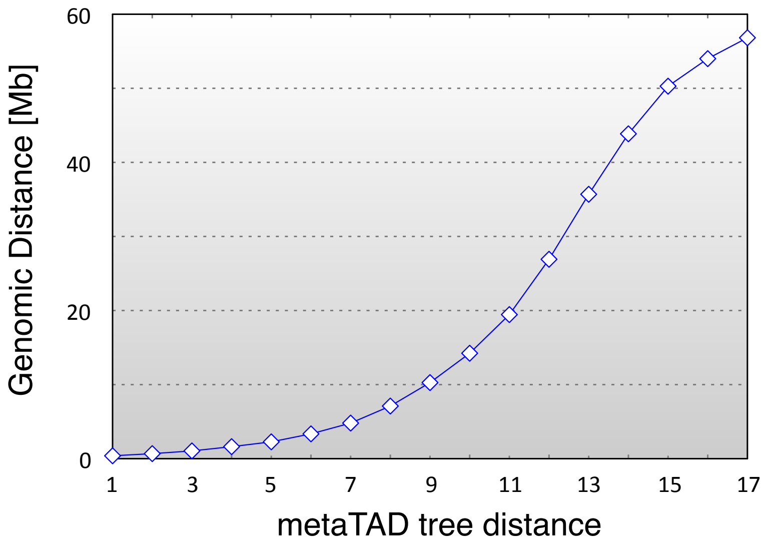

Appendix Figure S21. Relationship of metaTAD tree distance with genomic distance.
The plot shows the average genomic distance corresponding to a given metaTAD tree distance over the tree. Data are averaged over the three cell types of this study.

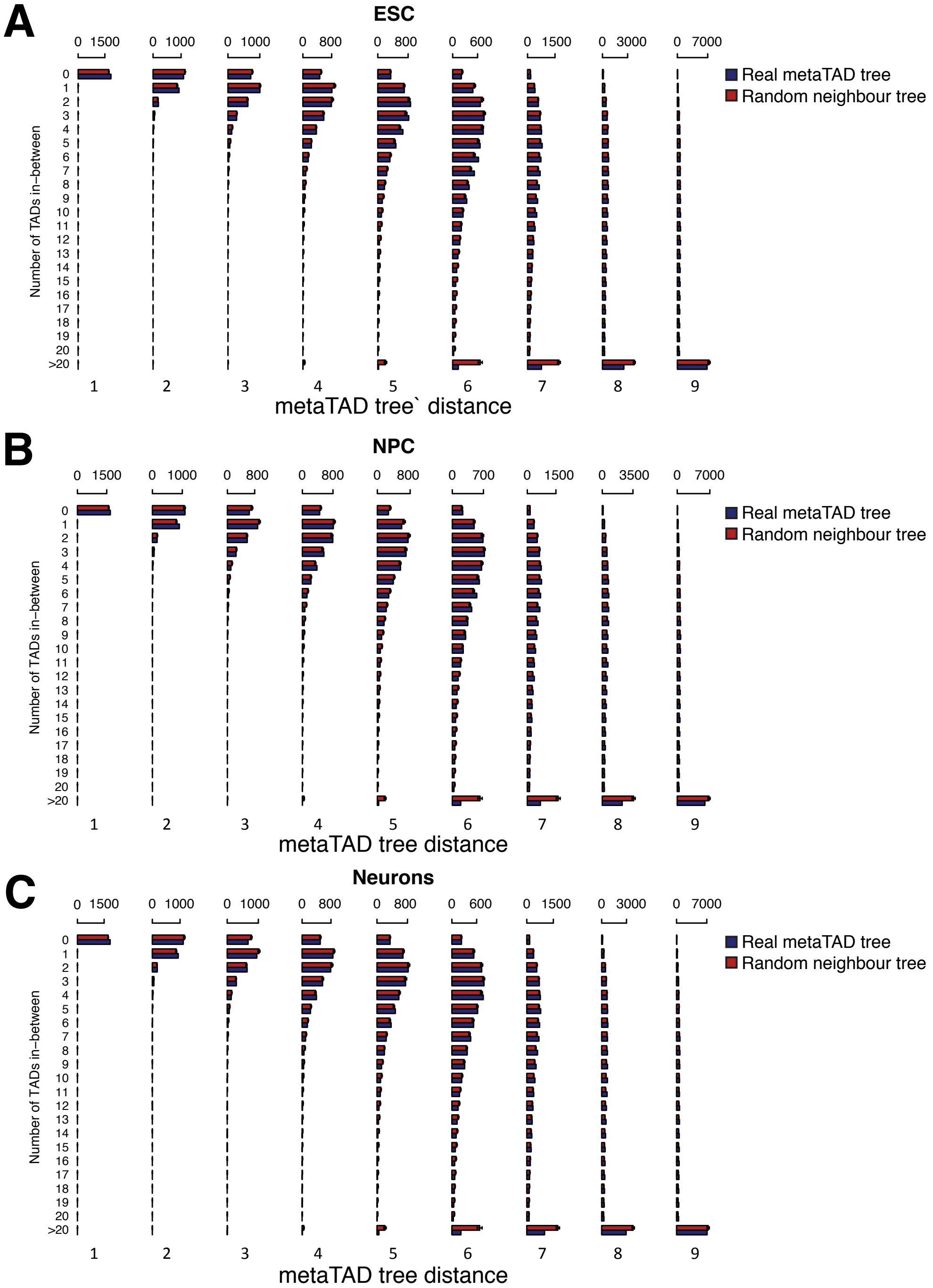

Appendix Figure S22. Distribution of metaTAD sizes.
(**A-C**) Histograms of linear distances between TADs at fixed metaTAD tree distances measured as the number of TADs lying in-between, for ESC (**A**), NPC (**B**) or Neurons (**C**). Two directly neighbouring TADs have a linear distance of 0 (no intermediate TAD). The distributions based on observed (blue) and random neighbour (red) trees show highly similar distances for all three time-points. Error bars reflect one standard deviation based on 30 random trees.

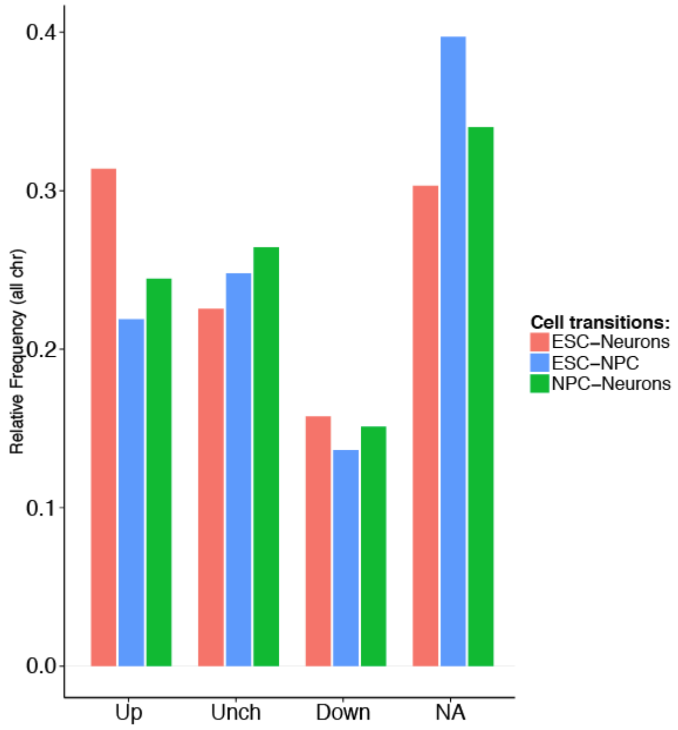

Appendix Figure S23. Classification of gene expression change of conserved TADs between time points.

Bar plot with the relative frequency of each class of gene expression change between the different differentiation time-points. NA refers to conserved TADs which did not fit the criteria for being up-regulated, down-regulated or unchanged (see Supplementary Methods).

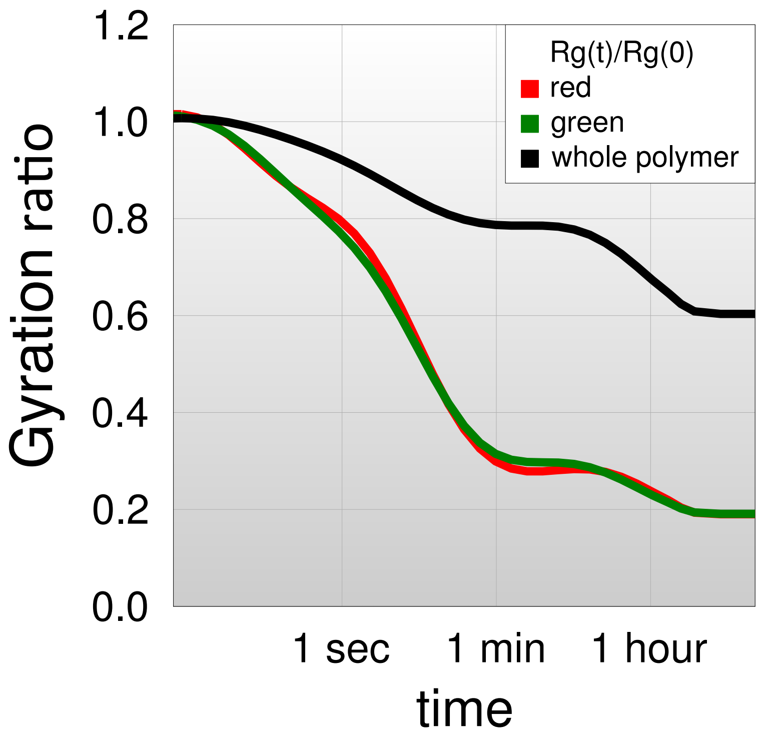

Appendix Figure S24. Monte Carlo equilibration dynamics of the SBS polymer model.
In our SBS polymer simulations, the system is left to equilibrate until it reaches a stable thermodynamics state. Here we show the equilibration dynamics of the normalized gyration radius *Rg(t)/Rg(0)* starting from open (SAW) interaction-free polymer configurations in the model of Fig 5B. The three curves refer to the whole polymer (black), and to only red or only green beads.

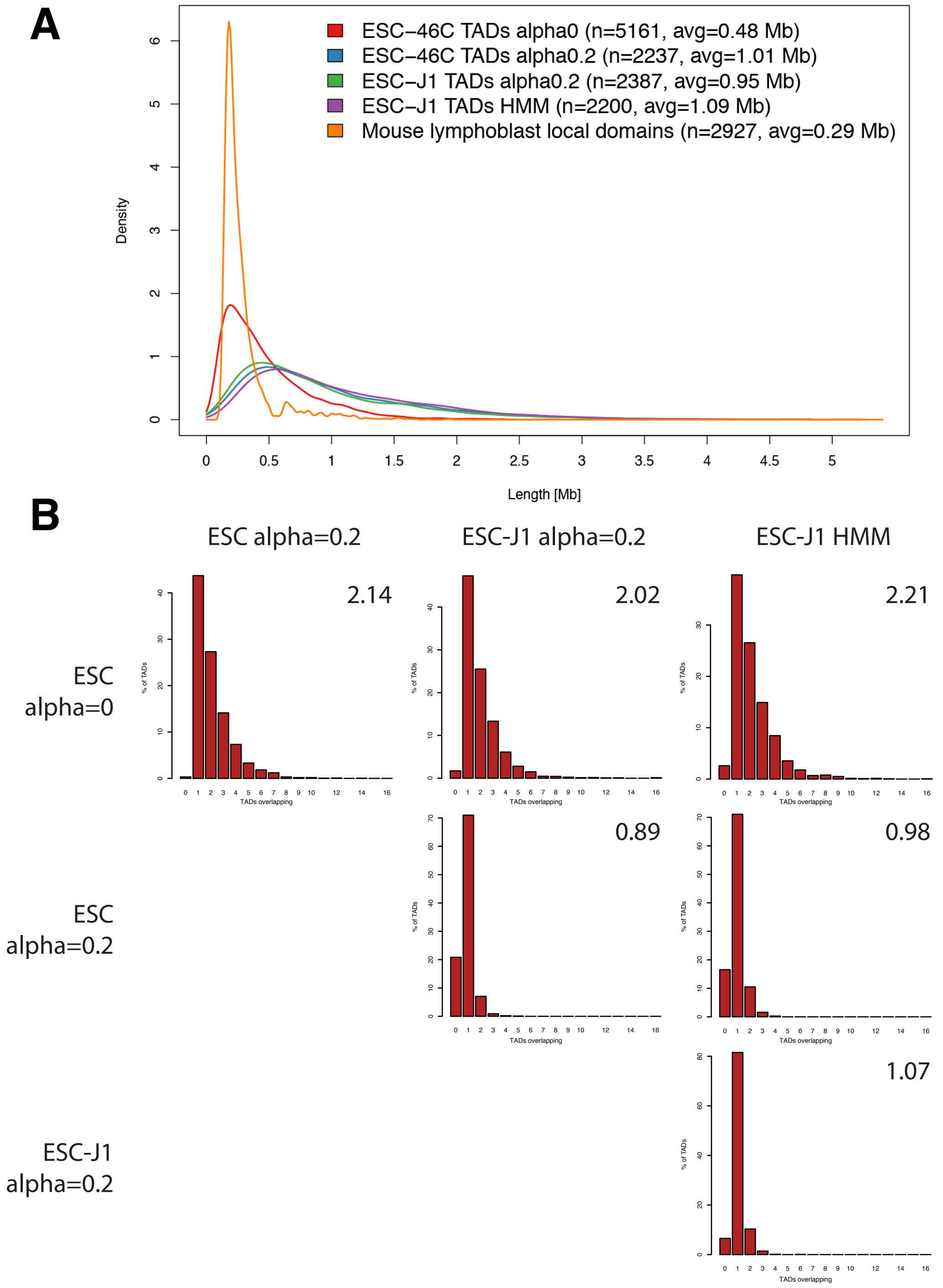

Appendix Figure S25. Comparing TAD definitions between different datasets and algorithms. (**A**) Density plot showing the distribution of TAD/domain sizes dependent on the used algorithm/ dataset. The ESC-J1 TADs HMM were published in Dixon *et al.* (2012), the mouse lymphoblast local domains were published in Rao *et al.* (2014). The TADs used in our study (ESC-46C, α = 0; red) have an average size of approx. 0.5 Mb. The number of TADs derived and their average length is shown in the upper right corner. (**B**) Histograms of the number of TADs from one algorithm/dataset (left) overlapping TADs based on another algorithm/dataset (top). Only TADs overlapping with >50% of their size were counted. The average count of overlapping TADs for each comparison is shown in the upper right corners.

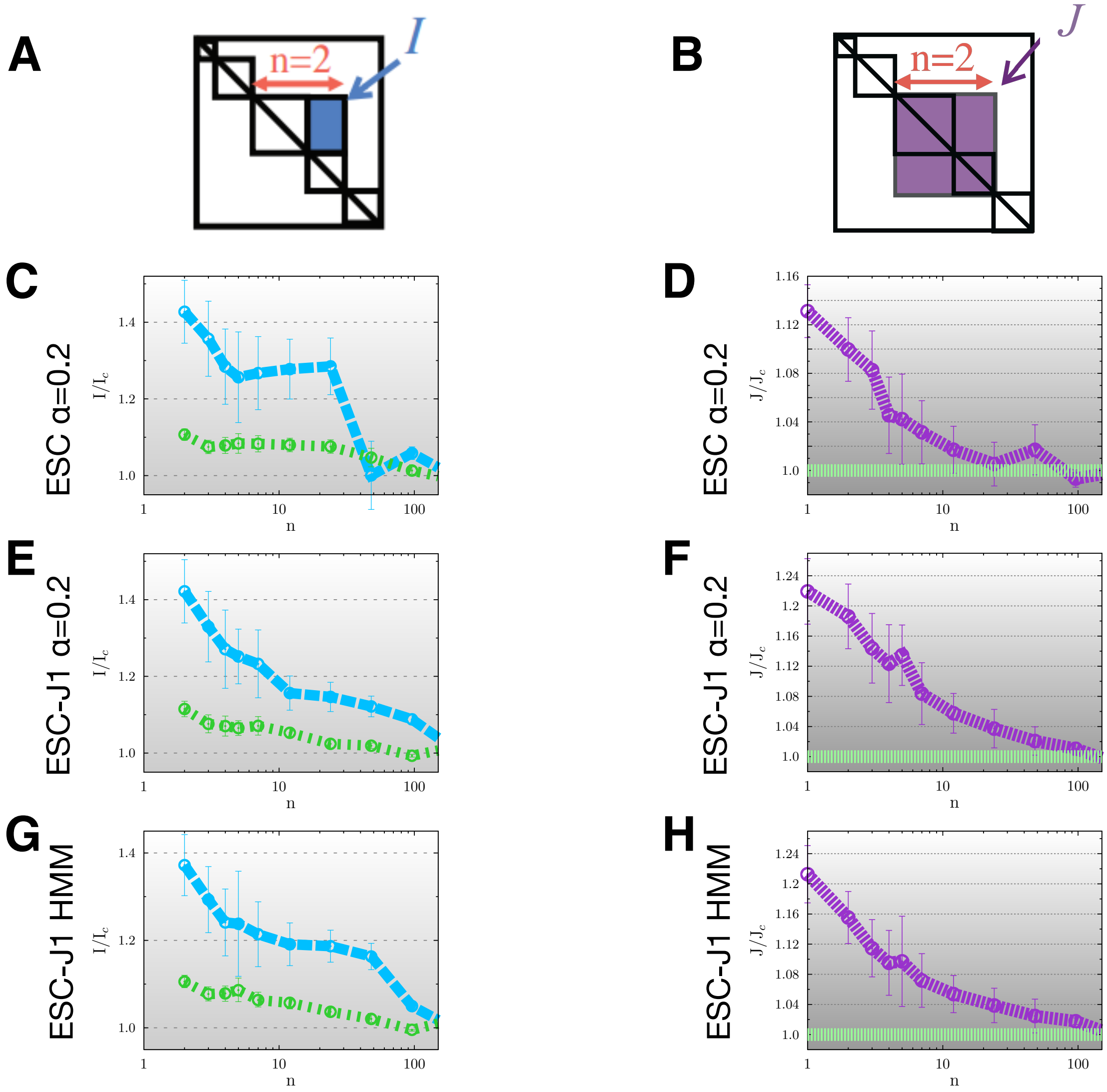

Appendix Figure S26. General features of metaTAD trees are significant and independent of the algorithm and dataset used.

The figure shows the genome averaged I/I_c (blue, **A**) and J/J_c (purple, **B**) curves, and the corresponding control cases (in green), in different murine Hi-C data. Panel (**C**) and (**D**) correspond to metaTADs in our ESC-46C data, using TADs identified with our method for α = 0.2. Panel (**E**) and (**F**) correspond to metaTADs in the original ESC-J1 data from Dixon *et al.* (2012), using TADs identified with our method at α = 0.2. Panel (**G**) and (**H**) correspond to metaTADs in the original ESC-J1 data from Dixon *et al.* (2012), using the original HMM TAD classification published in Dixon *et al.* (2012).

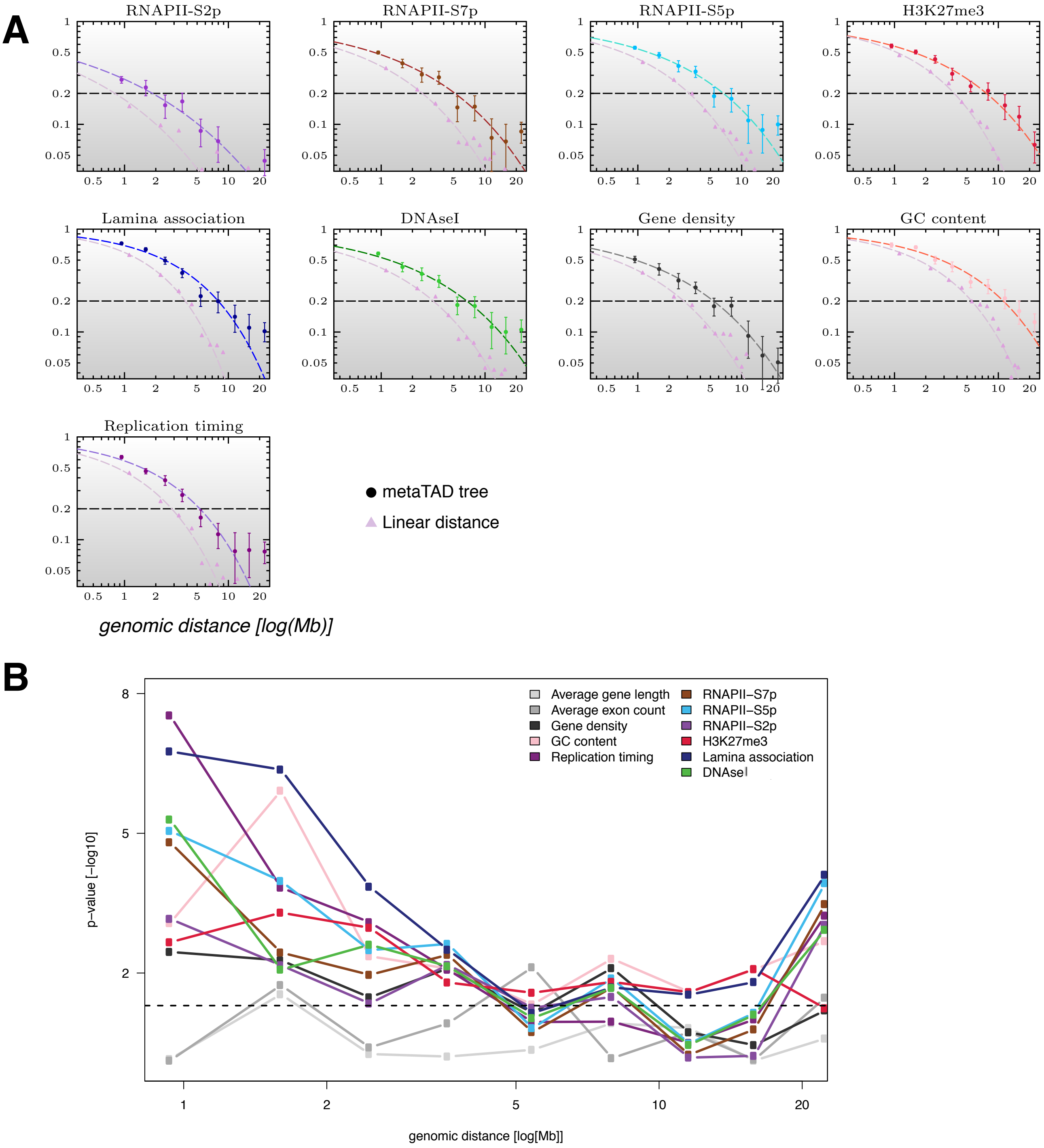

Appendix Figure S27. Correlations of genomic features along metaTAD trees derived using ESC TADs (α = 0.2) are much stronger than expected by linear genomic distances.
(**A**) Correlation coefficient of different epigenetic features over the metaTAD tree (filled circles, upper lines) in ESC data, using TADs identified with our method for α=0.2. MetaTAD tree correlations are larger than the same correlation measured over the linear genomic distance of TADs (filled pink triangles). (**B**) To measure significance, we computed one-sided Wilcoxon tests of difference in median between the real and random neighbour tree correlations and found the former to be significantly stronger (horizontal line, p-value=0.05) up to large genomic distances.
