# Supplementary material for: Hierarchical folding and reorganization of chromosomes are linked to transcriptional changes in cellular differentiation
Source: Mol Syst Biol. 2015 Dec 28;11(12):852. doi: 10.15252/msb.20156492 (PMC4704492; doi:10.15252/msb.20156492)
Supplement: Supplementary file 2 — Expanded View Figures PDF [file MSB-11-852-s002.pdf]

Expanded View Figures

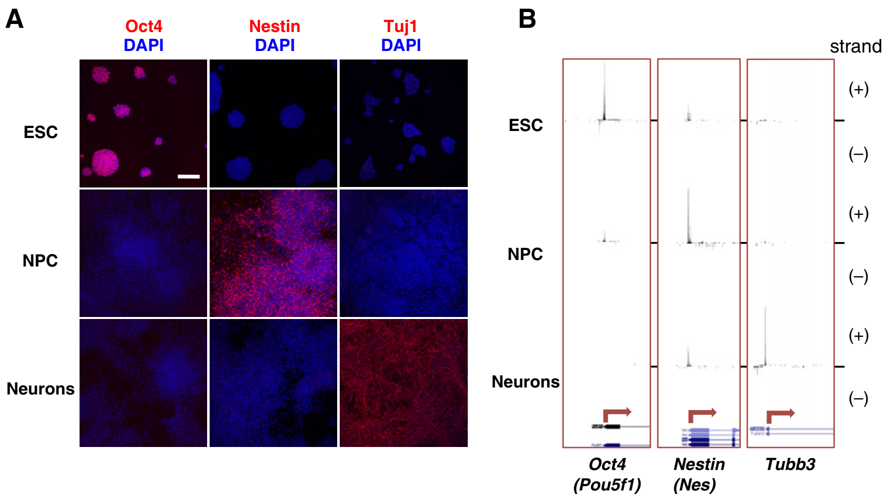

Figure EV1. Differentiation of mouse ESC (46C line) into midbrain-like neuronal cells.

A Cell populations were tested by immunofluorescence staining for their purity using stage-specific markers (pseudocoloured red), which showed efficient progression of ESC through the differentiation steps. *Oct4* expression in ESC is lost upon differentiation, *nestin* is specifically expressed in NPC, and *Tubb3* (detected using Tuj1 antibodies) is strongly expressed in Neurons. Nuclei were counterstained with DAPI (pseudocoloured blue). Scale bar represents 100 μm.

B Total RNA was extracted from ESC, NPC and Neurons, and directional CAGE data sets were produced in order to measure RNA transcription and define transcription start sites in each time point. Strand-specific CAGE reads are represented (+ and – strands). The promoter regions of the stage-specific markers are reported. CAGE signals for *Oct4* (*Pou5f1*), *nestin* (*Nes*) and *Tubb3* genes peak in ESC, NPC and Neurons, respectively.

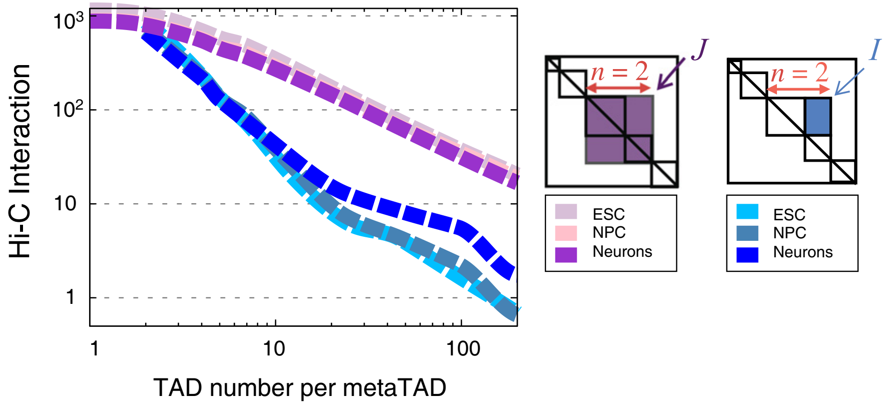

Figure EV2. Frequency of TAD–TAD interactions at each tree level.

The average metaTAD inter-domain, *I* (blue curves), and intra-domain, *J* (magenta), interactions are shown as a function of the number of TADs, *n*, which a metaTAD includes in our three cell types (see also Appendix Supplementary Methods). The larger the domains considered, the lower their average interactions, consistently with Hi-C results. As expected, intra-domain interactions, *J*, stay always above inter-domain interactions, *I*.

© 2015 The Authors

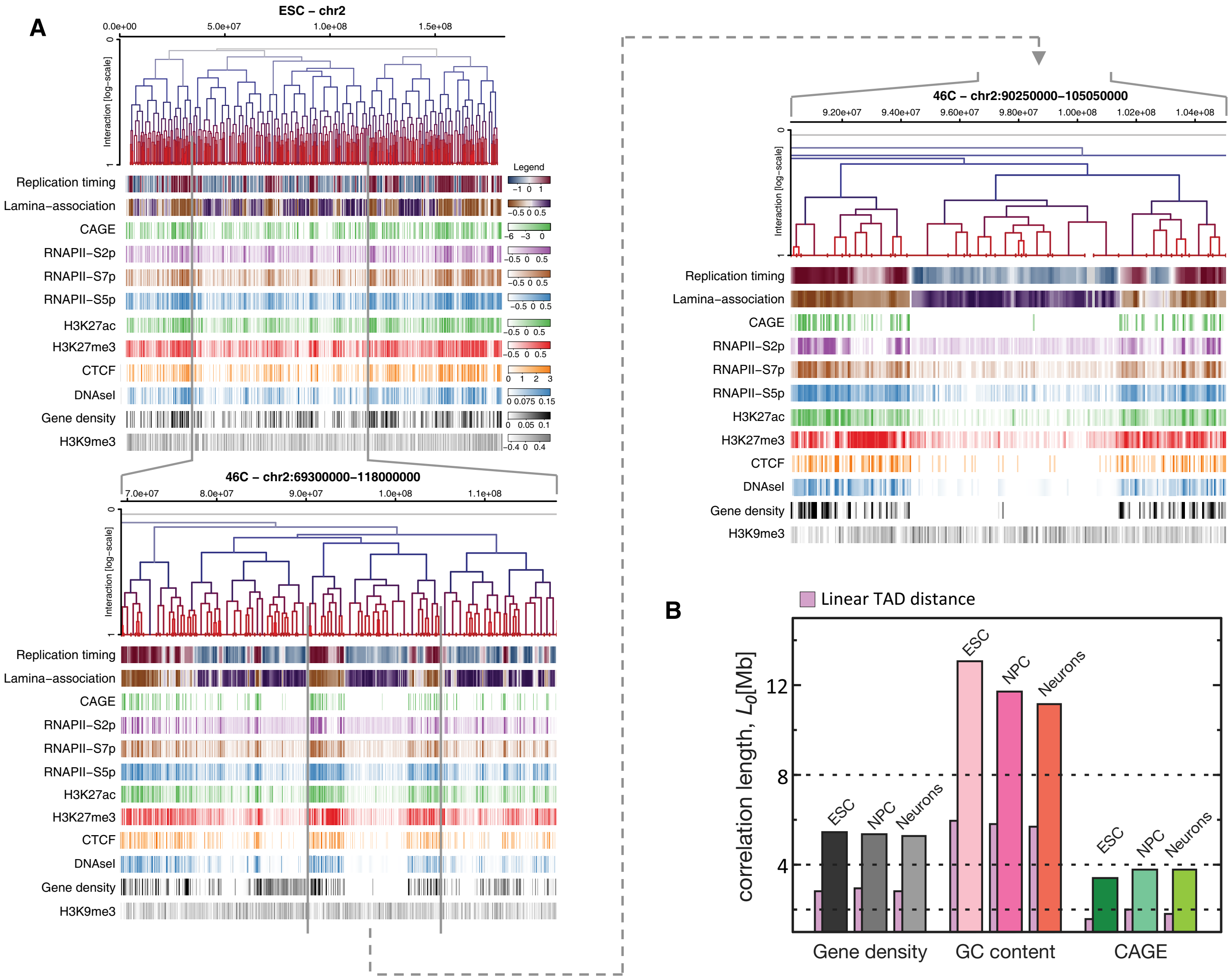

Figure EV3. Correlation of genomic, epigenomic and transcription factor features.

A Three nested levels of TAD hierarchies of chromosome 2 are displayed above multiple genomic, epigenomic, CAGE data and transcription factor (TF) features measured in ESC. Feature intensities are evaluated in 50-kb windows and have been clipped at 5 and 95% quantiles to facilitate visualization. The colour keys reflecting the full range of feature scores over all chromosomes are depicted on the right of the topmost panel.

B Correlation lengths measured across metaTAD trees in each time point (ESC, NPC and Neurons) were calculated for gene density, GC content and CAGE tags. Despite metaTAD restructuring (see Fig 2F), strong correlations are detected at each time point.

© 2015 The Authors

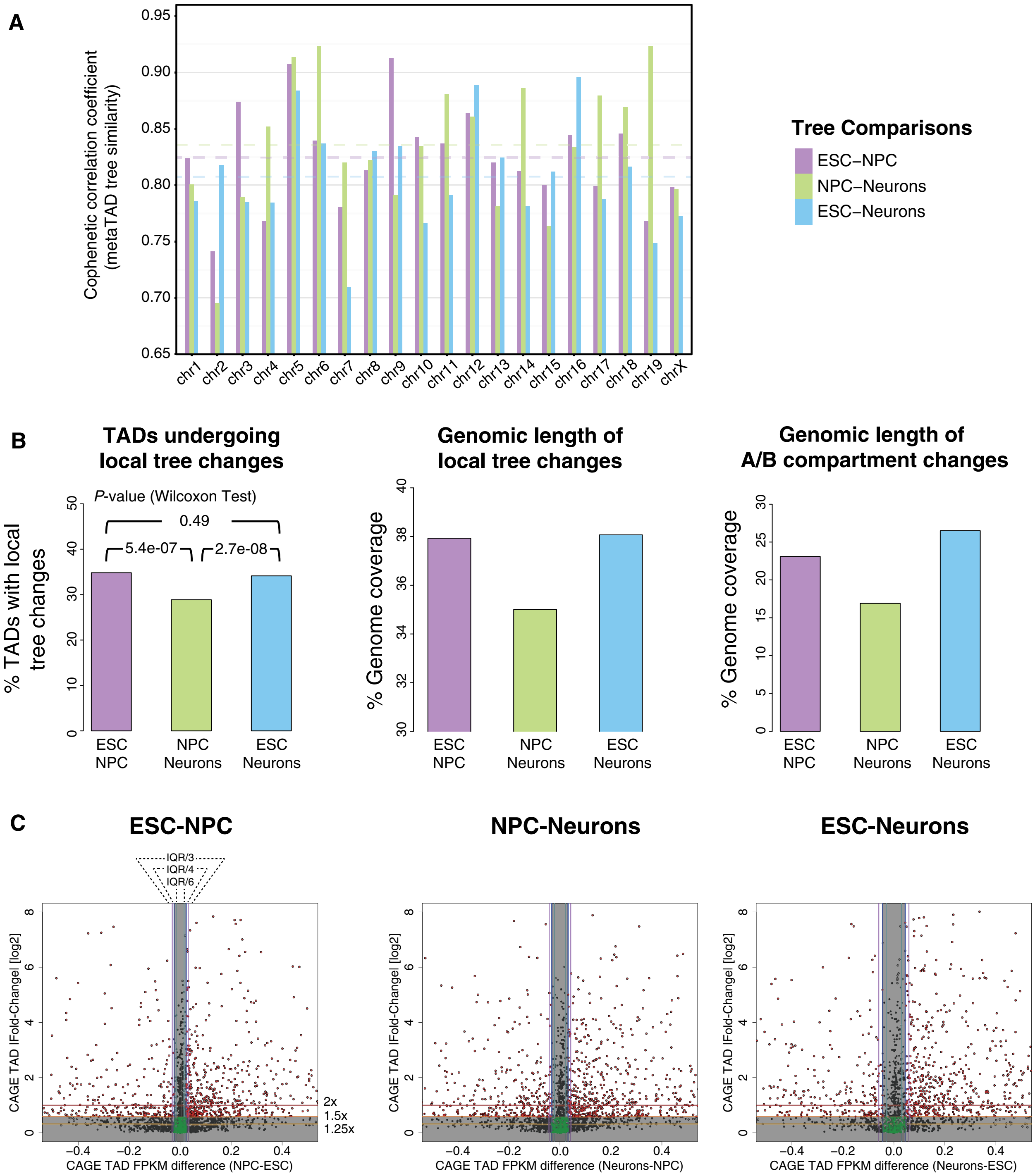

Figure EV4. Re-wiring of metaTAD trees across differentiation and TAD-based gene expression changes.

A Cophenetic correlation coefficients comparing metaTAD trees per chromosome show different levels of tree restructuring in each differentiation time point transition. Dashed horizontal lines represent the average value for all autosomal chromosomes.

B Percentage of the total number of (left to right): TADs with a local tree change value above a threshold of 1 (threshold values of 0.5, 2 or 3 for local tree change gave similar results; *P*-values refer to Wilcoxon rank-sum test performed with all tree change values in each time point transition); genomic extension of regions characterized as tree change (*z*-score > 0); and genomic extension of regions characterized as A/B compartment change (> 25% coverage change) for all time point transitions.

C Scatter plots illustrating TAD classification according to the gene expression change double criteria used in Fig 4: fold change (> 1.5×) and FPKM difference (> IQR/4). Other thresholds considered are also marked. TADs in red are significantly changing, while TADs in green do not display significant changes in expression.

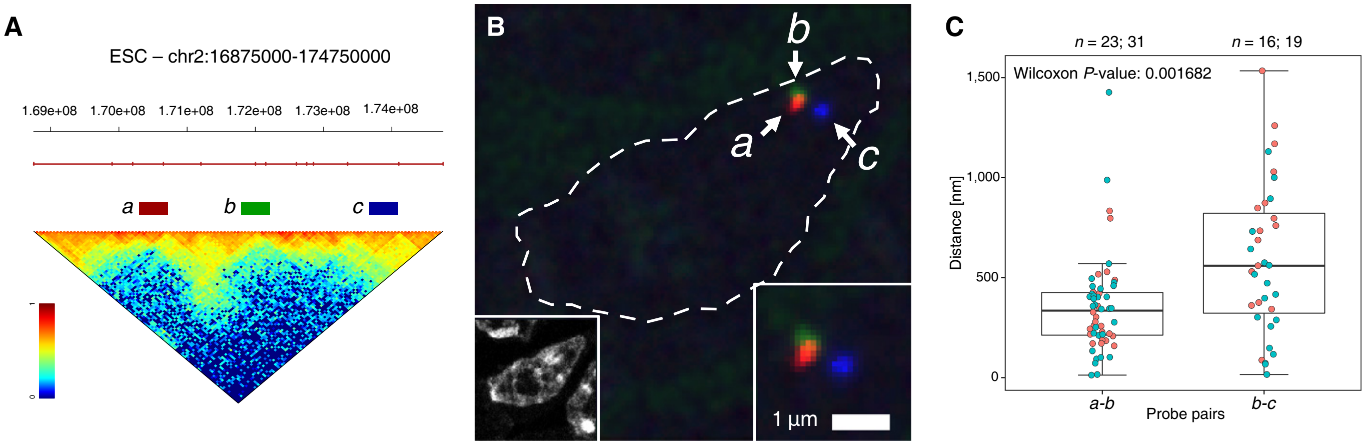

Figure EV5. Long-range contacts detected by Hi-C between distant TADs can be confirmed at the single-cell level by cryoFISH.

A To assess the extent long-range interactions between TADs at the single-cell level, we designed three oligo libraries tagged with ATTO-550, ATTO-488 and ATTO-594, which cover genomic regions *a* (red), *b* (green) and *c* (blue), separated by 1.5 (*a-b*) or 2 (*b-c*) Mb, and which belong to different TADs (red line). Hi-C data show stronger long-range interaction frequency between *a* and *b* than *b* and *c*.

B Probes *a*, *b* and *c* were simultaneously hybridized on ultrathin (~180-nm-thick) cryosections from ESC (Branco & Pombo, 2006), before counterstaining with DAPI (inset on the left) and imaged on a confocal microscope. Inset on the right shows a magnified region of the same image. Dashed line indicates the nuclear outline. Region *b* often co-localizes with region *a*, but not region *c*.

C Distances between regions *a* and *b*, or *b* and *c*, were measured using an automated macro from the centroids of fluorescent signals corresponding to *a*, *b* and *c*. Data were collected from two independent cryoFISH experiments (red and green dots); number of distances is indicated above the graph. The average distance between the centre of the fluorescent signals corresponding to probes *a* and *b* is 350 ± 242 nm, in contrast with probes *b* and *c*, which are on average separated by 587 ± 375 nm (Wilcoxon test, *P*-value 0.00168). The ratio of the genomic separation for the two pairs of probes (*b-c*/*a-b*) is ~1.3, compared with their physical distance ratio of 1.7.

 © 2015 The Authors
